# Knowledge and perception of dementia risk and protective factors: a systematic review and meta-analysis

**DOI:** 10.1016/j.tjpad.2026.100565

**Published:** 2026-04-11

**Authors:** Muhammed L. Sambou, Jolanda H.M. Dobbe, Elaine A.C. Albers, Kay Deckers, Lidia R. Arends, M. Arfan Ikram, Ellen M.A. Smets, Wichor M. Bramer, Jeremy A. Labrecque, Leonie N.C. Visser, Frank J. Wolters

**Affiliations:** a Department of Epidemiology, Erasmus MC University Medical Center, Rotterdam, the Netherlands; b Department of Medical Psychology, Amsterdam University Medical Center location AMC, University of Amsterdam, Amsterdam, the Netherlands; c Alzheimer Center Amsterdam, Neurology, Vrije Universiteit Amsterdam, Amsterdam UMC location VUmc, Amsterdam, the Netherlands; d Amsterdam Public Health, Quality of Care, Amsterdam, the Netherlands; e Amsterdam Public Health, Ageing and later life, Amsterdam, the Netherlands; f Alzheimer Centre Limburg, Department of Psychiatry and Neuropsychology, Mental Health and Neuroscience Research Institute (MHeNs), Maastricht University, Maastricht, the Netherlands; g Department of Biostatistics, Erasmus Medical Center, Rotterdam, the Netherlands; h Amsterdam Public Health, Personalized medicine, Amsterdam, the Netherlands; i Medical Library, Erasmus MC University Medical Center, Rotterdam, the Netherlands; j Division of Clinical Geriatrics, Center for Alzheimer Research, Department of Neurobiology, Care Sciences and Society, Karolinska Institutet, Stockholm, Sweden; k Department of Bioethics and Health Humanities, Julius Center for Health Sciences and Primary Care, University Medical Center Utrecht, Utrecht University, Utrecht, the Netherlands; l Department of Radiology & Nuclear Medicine and Alzheimer Center, Erasmus MC University Medical Center, Rotterdam, the Netherlands

**Keywords:** Primary prevention, Public health, Education, Behaviour, Environment, Cardiometabolic risk factors

## Abstract

**Background:**

Optimal dementia risk reduction strategies benefit from sufficient public knowledge of risk factors and risk perception, but current public awareness is uncertain.

**Methods:**

In a systematic literature review on public knowledge and perception of dementia risk factors, we searched relevant databases for original research articles until 2024. When possible, we pooled study results using random effects meta-analysis, and explored sources of heterogeneity through meta-regression. Qualitative studies and studies about risk perception were analysed using narrative synthesis.

**Findings:**

Of 4996 articles screened, 155 were eligible for inclusion. Of these, 125 reported on knowledge of risk factors and 50 on risk perception, jointly providing data from 164,644 participants in 41 countries across 6 continents. Recognition of the 28 queried risk and protective factors was moderate, somewhat higher for lifestyle factors (medians: 38.5–71.5%) than for cardiovascular (9.9–66.9%) and environmental (25.4–44.4%) factors, but with large heterogeneity across queried factors. With the exception of physical activity (71.5%, IQR:46.9–88.3%), social isolation (66.6% [23.7–84.0%]) and traumatic brain injury (65.0% [18.0–76.7%]), recognition of all established modifiable risk factors for dementia from prespecified lists was below 50%, lowest for education (19.5% [7.8–54.9%]), air pollution (25.4% [16.3–41.0%]), and obesity (30.4% [27.0–43.0%]). Recall of risk factors (7 studies) was markedly lower than recognition. Meta-regression analyses showed no consistent differences by year of publication, or by participants’ age, gender, and educational attainment. Among 23 qualitative studies, limited knowledge emerged particularly regarding dementia-specific risk factors like hearing loss. Perceived risk was measured inconsistently across studies, but was generally moderate to high, along with notable worry about dementia in a large part of the older population.

**Conclusions:**

Knowledge of dementia risk and protective factors in the general population remains limited. These findings call for population-level interventions, including educational campaigns, to enhance preventive strategies.

## 1 Background

At present, more than 55 million people are living with dementia, posing a burden on individuals, families, and societies worldwide [[1], [2], [3], [4]]. About 45% of these dementia cases are attributable to 14 modifiable risk factors, ranging from factors in early life (e.g., less education) to late-life visual loss and social isolation [5]. In addition, several other emerging risk and protective factors have gained attention as a potential target for prevention, including stress, sleep disturbances, pesticide exposure, low cognitive activity and unhealthy diet [[6], [7], [8], [9], [10], [11]]. Whilst these findings underline the potential of dementia prevention, risk factor reduction remains challenging, and the prevalence of certain risk factors like obesity and diabetes is increasing [12]. The vast majority of dementia prevention initiatives target risk factors through individual-level interventions and behavioural change [13]. Awareness and knowledge among the general population and healthcare professionals is a prerequisite for the sustained effect of these individual-level interventions, and the broader uptake of brain healthy behaviours in the population [[13], [14], [15], [16]]. Moreover, corresponding changes in risk behaviour are largely determined by risk awareness – i.e., a person’s belief about their susceptibility and severity of an event [1,[17], [18], [19], [20]].

Over the past few years, attention for dementia risk reduction in the media has increased and public education campaigns have been developed to improve knowledge [[21], [22], [23], [24].] However, it remains uncertain whether this has translated into increased knowledge, as a comprehensive overview of public risk awareness is lacking. Prior systematic reviews on this topic, including data up till 2014, 2017 and 2019, reported an increase in the awareness that dementia can be a preventable disease, but knowledge of the risk and protective factors of dementia was only fair to moderate [[25], [26], [27]]. These reviews mainly focused on non-modifiable factors (e.g. age, gender, genetics) and a selection of lifestyle factors, leaving insight on more recent modifiable factors (e.g. hearing loss, vision loss) unaddressed. Furthermore, variation in knowledge between geographical regions and subgroups of the population could guide educational interventions, but such variation has not been systematically mapped beyond that in cardiovascular risk factors [27].

Therefore, we aimed to synthesise current evidence on global perception of dementia risk, and knowledge of its risk and protective factors through a systematic review and meta-analysis of the literature.

## 2 Methods

### 2.1 Search strategy

Together with an experienced librarian (WMB), we developed a search strategy, covering a combination of terms (see Supplemental File 1 for the full searches). Five databases were included: MEDLINE ALL via Ovid, Embase via embase.com, Web of Science Core Collection, Cochrane Central Register of Controlled Trials via Wiley, and PsycINFO via Ovid. Both a generic search term and specific risk factors were included (based on relevant literature), thereby including all known and presumed risk/protective factors of dementia, irrespective of the robustness of their potential causal link to dementia. Regarding risk perception, our search included both direct assessment (e.g., ‘perceived risk of developing dementia later in life’) and indirect assessment methods (e.g., perceived severity, worry, concern, fear, and related terms). The search spanned from database inception till January 4th, 2024 (see Supplemental File 1 for start dates per database). This report follows the Preferred Reporting Items for Systematic Reviews and Meta-Analyses (PRISMA) guideline [28]. The protocol is registered at the PROSPERO database (ID: CRD420250636873).

### 2.2 Inclusion and exclusion criteria

We included English language peer-reviewed articles containing original empirical data, either quantitative or qualitative, on knowledge regarding dementia risk- and protective factors and/or risk perception. Both hospital- and population-based studies were included, and there was no restriction on geographical location or study design. We excluded articles that:

a) Reported solely about beliefs of dementia preventability, attitudes towards dementia, attitudes towards ageing, and beliefs about dementia being a normal part of ageing;
b) Primarily focused on people with dementia. When a part of the study population concerned people with dementia and this group was considered separately within the data analyses, the article was still included;
c) Reported only total scores or subdomain scores of a scale to assess knowledge of risk and protective factors rather than the individual items, or;
d) Reported no baseline knowledge on dementia risk- and protective factors and/or risk perceptions in case of educational intervention studies (e.g. e-module or public health campaigns on dementia risk reduction).

### 2.3 Study selection and data extraction

Article citations were imported and deduplicated in EndNote [29]. Screening on title, abstract and full-text was performed by two independent reviewers (MLS and JHMD), using Covidence, a web-based collaboration software platform for systematic reviews [30]. Conflicts were resolved through consensus discussion by the two reviewers. A data extraction form was developed, including authors, study aims, other study characteristics (see Table 1), used scales, and results. Data were extracted by MLS and JHMD verified whether these corresponded to the original source material. Corresponding authors were contacted via email if data was not available or partly missing.

**Table 1:** Descriptive characteristics of all 155 included articles.

Variables	Knowledge of risk and protective factors (N = 125*)		Risk perception (N = 50*)
	Quantitative (N = 103)	Qualitative (N = 23)	
Sample size, median [IQR]	551 [223 – 1162]	28 [20 – 40]	323 [164 – 1071]
Age, mean ± SD †,‡	47.0 ± 14.8	55.0 ± 13.2	54.2 ± 15.8
Female, mean % ± SD †	60.4 ± 12.3	61.7 ± 12.1	59.3 ± 11.0
Study population, n (%) §			
Adult general population	73 (70.8)	16 (69.6)	39 (78.0)
Healthcare professionals	18 (17.5)	3 (13.0)	0
Caregivers of people with dementia	10 (9.7)	4 (17.4)	8 (16.0)
Other	10 (9.7)	4 (17.4)	9 (18.0)
Education †, ¶			
Education in years, n (%)	9 (8.7)	2 (8.7)	8 (16.0)
Number of years, median [IQR]	11.8 [8.9 – 12.9]	13.3 [12.6 – 13.9]	14.0 [13.8 – 14.8]
Education in categories, n (%)	87 (84.5)	12 (52.2)	33 (66.0)
Low, mean % ± SD	14.3 ± 19.1	22.7 ± 24.5	18.6 ± 17.9
Further, mean % ± SD	28.3 ± 21.9	24.7 ± 15.8	31.7 ± 17.1
Higher, mean % ± SD	56.4 ± 28.8	48.9 ± 23.5	49.1 ± 24.2
Geographical location, n (%)			
North America	22 (21.4)	4 (17.4)	15 (30.0)
South America	6 (5.8)	0	1 (2.0)
Europe	25 (24.3)	12 (52.2)	14 (28.0)
Asia	34 (33.0)	4 (17.4)	13 (26.0)
Africa	5 (4.9)	1 (4.3)	0
Oceania	11 (10.7)	2 (8.7)	5 (10.0)
Multiple continents	0	0	2 (4.0)
Year of publication, n (%)			
≤ 2015	18 (17.5)	3 (13.0)	15 (30.0)
2016 – 2020	37 (35.9)	7 (30.4)	15 (30.0)
≥ 2021	48 (46.6)	13 (56.5)	20 (40.0)
Sampling method, n (%) #			
Non-probability	78 (75.7)	23 (100)	42 (84.0)
Probability	25 (24.3)	0	8 (16.0)
Recruitment method, n (%) †			
Direct approach (in the community)	55 (53.4)	18 (78.3)	26 (52.0)
Through telephone/ online	44 (42.7)	2 (8.7)	21 (42.0)
Mixed	4 (3.9)	2 (8.7)	3 (6.0)
Assessment method, n (%)			
Self-administered	83 (80.6)	0	31 (62.0)
Interview-administered	16 (15.5)	10 (43.5)	16 (32.0)
Focus group discussion	0	8 (34.8)	2 (4.0)
Mixed	4 (3.9)	5 (21.7)	1 (2.0)

Notes.

⁎ Some articles are part of more than one group (e.g., articles with a mixed-methods quantitative and qualitative design), and therefore the total number of included articles is lower than the sum of the three groups. Articles about risk perception were not stratified in quantitative and qualitative articles as there were only 4 qualitative articles.

† Missing values: quantitative studies on knowledge: age (n = 5), gender (n = 3) and education (n = 7); qualitative studies on knowledge: age (n = 6), gender (n = 3), education (n = 9) and recruitment source (n = 1); studies on risk perception: education (n = 9).

‡ For studies reporting the population age in categories only (41/155 studies), median age was approximated based on the age distribution in the categories (for an example, see Supplemental File 2).

§ Sum of percentages exceeds 100% as several studies reported on different types of populations. “Healthcare professionals” include general practitioners, pharmacists, practice nurses and medicine students. “Other” includes students without a medical background, policymakers, religious leaders, and persons without dementia who were admitted to the hospital.

¶ The percentage of participants having low, further and higher education was calculated per article, after which the mean and SD across all articles was calculated.

# Non-probability sampling includes quota, purposive and convenience sampling. Probability sampling includes random, stratified and systematic sampling.

### 2.4 Quality assessment

We evaluated the quality of included articles using The Joanna Briggs Institute (JBI) Critical Appraisal Checklist for studies reporting prevalence data, and the JBI Critical Appraisal Checklist for Quantitative studies (Supplemental File 3 and 5) [31,32]. Quality assessment for each article was done by one researcher (JHMD), with adjudication by a second researcher (EACA).

### 2.5 Statistical analysis

Across all quantitative studies on knowledge of risk and protective factors, we determined the percentage of participants who correctly identified each factor to be a risk or protective factor for dementia (dubbed ‘correct knowledge’), and summarized these into a minimum, median, first quartile (Q1)–third quartile (Q3) and maximum. If data were sufficiently comparable, we pooled results using random effects meta-analysis. We further selected eight predictor variables for weighted meta-regression analysis: mean/median age (continuous), gender (% women), education (% with higher education), quality appraisal score (continuous), study population (adult general population, health professionals, other), year of publication (≤2015, 2016–2020 and ≥2021), geographical location (categories), and assessment method (self-administered, interview-assisted questionnaires, or combined). Missing values for predictor variables (age [7.1%], gender [3.0%], and education [15.2%]) were imputed using multiple imputation by chained equations (‘mice’) with predictive mean matching. The meta-regression was performed for all risk and protective factors that were reported by at least 20 studies, including a univariable model, and a model adjusting for age, gender, education, and study population [33]. If meta-regression showed signs of effect modification across at least three risk factors, results were presented stratified by subgroup. In addition, funnel plots and Egger’s regression tests were conducted to assess heterogeneity in study designs. All quantitative analyses were performed using R software (version 4.3.2; packages: mice, tidyverse, ggplot2, gmodels), with alpha set at 0.05 (two sided testing).

Articles reporting qualitative data on knowledge of risk and protective factors were described narratively, based on the narrative synthesis framework [34,35]. One investigator (MLS) summarized relevant information per article, after which a second investigator (JHMD) searched for similarities and differences across studies. Articles about risk perception (including both quantitative and qualitative data) were combined to assess their methodological characteristics and mean levels of risk perception in a descriptive way.

## 3 Results

### 3.1 Study selection and characteristics

**Fig. 1:**
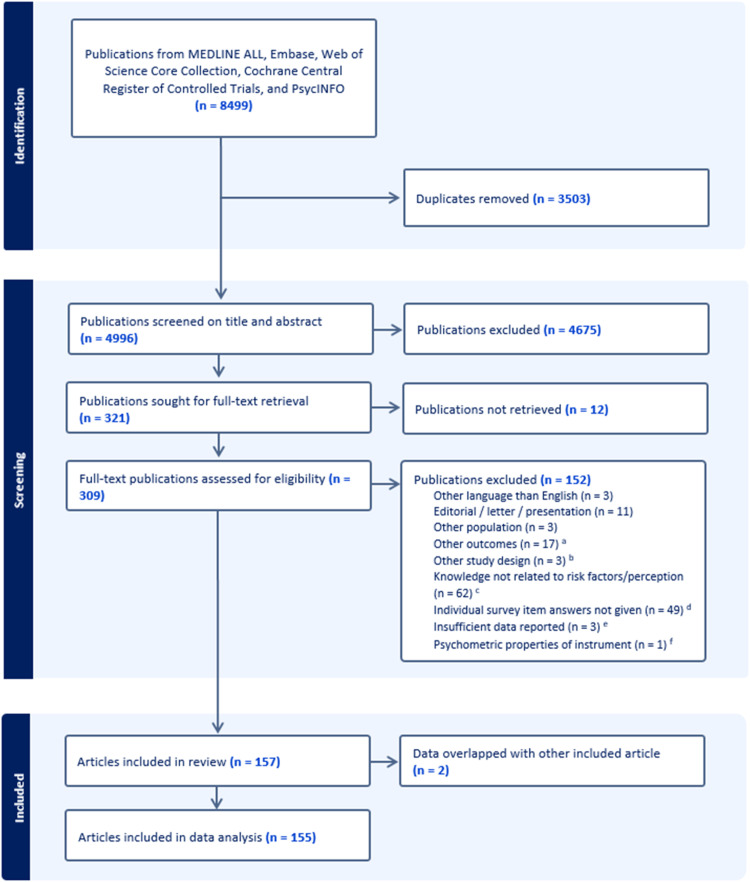
PRISMA flowchart. ^a^ This included outcomes like cognitive ageing, neurological diseases or brain health. ^b^ Two articles only measured awareness post intervention, and one article only described the need to measure misconceptions about dementia risk factors rather than measuring these beliefs. ^c^ The articles addressed knowledge of dementia in general, for example related to symptoms, attitudes towards dementia and dementia being part of the normal ageing process, but not related to risk factors or risk perception. For 7 of these articles, there was some data but this data was very limited. ^d^ Only the total score on an instrument, its subscale, or a combination of risk factors was provided. ^e^ Insufficient data were available for inclusion, despite contacting authors of the original publication. ^f^ This concerns an article that assessed the properties of an instrument, rather than the answers on that instrument.

**Fig. 2:**
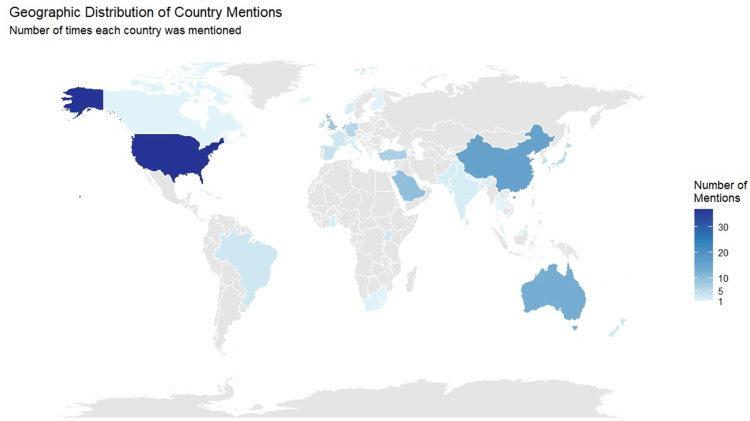
Geographical contributions to data presented in the systematic review. Legend: This world map shows the geographical location of all studies included in this review (N = 155).

Of the 4996 identified citations, 321 publications were sought for full text appraisal, of which 155 articles were included (Fig. 1). Of these, 125 examined knowledge of dementia risk- and protective factors (103 quantitatively, 23 qualitatively, one mixed-methods). Fifty studies reported data about risk perception (45 quantitatively, four qualitatively, one mixed-methods). Table 1 shows characteristics of the included studies. Jointly these provided data from 164,644 participants, mostly from the general public, and originating from all continents (Fig. 2). The number of publications increased over time, with 85 (82.5%) of quantitative and 20 (86.9%) of qualitative risk factor studies published since 2015. Nearly all studies had a cross-sectional design; for the two intervention studies, baseline parameters of knowledge were depicted.

### 3.2 Quality assessment

The full quality assessment is displayed in Supplemental File 3–6. Of the quantitative reports, the majority (117/131, 89.3%) had an appropriate sampling frame to address its target population, but only 28/131 (21.4%) articles used an appropriate sampling method (i.e., probability sampling). In 94/131 (71.8%) articles, response rates were low or not mentioned. Overall, 65/131 (49.6%) studies used valid methods for measuring knowledge or risk perception (i.e., (items copied from) validated questionnaire). In all qualitative articles, the research methodology was congruent with the research objective, participant voices were adequately presented, and conclusions seemed accurate. Yet, in 14/23 (60.9%) qualitative studies, the theoretical perspective was not indicated and the influence of the researcher on the research process was not discussed.

### 3.3 Knowledge of dementia risk and protective factors

#### 3.3.1 Quantitative studies

Overall, 28 risk factors were enquired in all cross-sectional studies, which assessed a median of three risk factors per article (IQR: 2–7). The vast majority of studies assessed knowledge by presenting participants with a list of predefined, potential risk factors (i.e., “recognition”; n = 94/103, 91.3%), whereas 9/103 studies (8.7%) asked participants to actively come up with factors (i.e., “recall”). For the 3 articles reporting both recognition and recall, we reported recall. Most studies used custom-made, study-specific questionnaires (54/103, 52.4%). Other used instruments included the Alzheimer’s Disease Knowledge Scale (ADKS; 25/103 studies, 24.3%), the Dementia Knowledge Assessment Scale (5/103, 4.9%), or other validated questionnaires (19/103, 18.4%; all used ≤ three times). Knowledge was generally quantified as the percentage of participants who identified a certain risk factor (97/103, 94.2%), with the remaining articles reporting ordinal categories (n = 5) or the total number of correctly identified risk factors (n = 1). In terms of risk factors studied, genetic predisposition and heritability were most often inquired (33/96 studies; 34.4%; Supplementary Table S7). Few studies assessed knowledge of environmental factors, with air pollution being included in 10 studies and pesticide exposure in 2 studies.

**Fig. 3:**
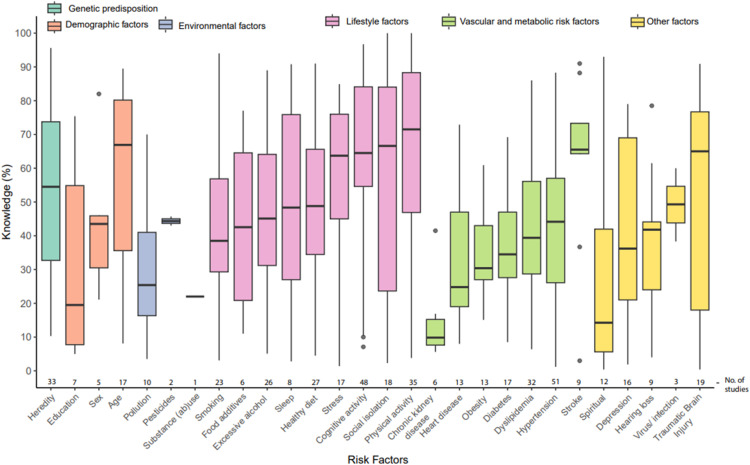
Knowledge of dementia risk and protective factors. Legend: Boxplots depict the median, along with the 25th and 75th percentiles; whiskers mark the 1st and 99th percentile, with outliers depicted as individual data points.

Fig. 3 shows overall recognition across different categories of risk factors (for precise percentages, see Supplementary Table S7). Genetic predisposition was most often mentioned by participants (median: 54.5%), followed by lifestyle factors (51.2%, range 38.5–71.5), demographics (43.3%, range 19.5–66.9), vascular and metabolic factors (35.5%, range 9.9–65.5) and environmental factors (34.9%, range 25.4–44.4). Among the individual non-modifiable factors, heredity and family history were identified by over half of participants on average (median: 54.5%, IQR: 32.7–73.8). Of all acknowledged modifiable risk factors for dementia, all but three were identified by fewer than 50% of the population, with lowest knowledge for less education (19.5%, IQR: 7.8–54.9), air pollution (25.4%, IQR: 16.3–41.0) and obesity (30.4%, IQR: 27.0–43.0) [5]. Recognition of established risk factors was highest for physical activity (71.5%, IQR: 46.9–88.3), social isolation (66.6%, IQR: 23.7–84.0) and traumatic brain injury (65.0%, IQR: 18.0–76.7). Having had a stroke, stress, and cognitive activity were also often mentioned to affect dementia risk (Fig. 3). Not many people indicated chronic kidney disease, substance abuse, or spirituality as risk factors for dementia.

Among nine studies that assessed recall, rather than recognition, similar types of risk factors were mentioned as with recognition, but knowledge was substantially poorer. This is summarised for the seven general population samples in Supplementary Table S8. Of the 28 risk factors that were investigated for recognition, 21 (75.0%) were mentioned at least once during recall. Of all 14 acknowledged modifiable risk factors, three were mentioned across all studies (i.e., excessive alcohol, physical activity, and social isolation) and four were not mentioned at all (i.e., education, obesity, diabetes, and hearing loss). With the exception of age, stroke and heart disease, recall for all risk factors was about 2 to 5-fold lower than in the studies on recognition. For example, smoking was recognised by a median 38.5% of participants, but only 24.0% mentioned smoking on recall. Similar examples included physical activity (71.5% versus 31.3%) and traumatic brain injury (65.0% versus 6.5%).

**Fig. 4:**
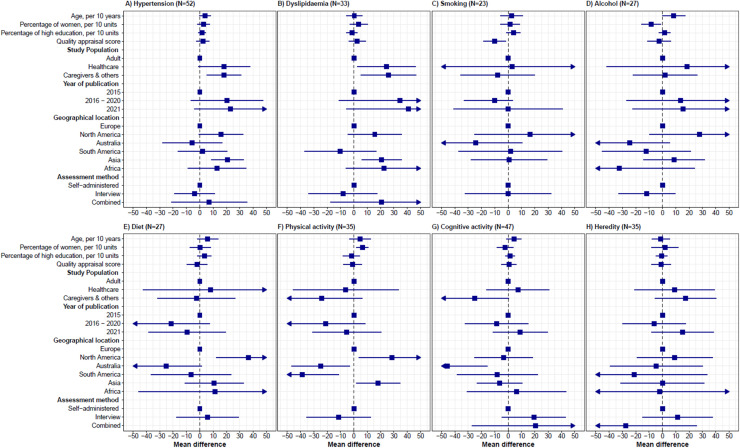
Study-level determinants of knowledge of dementia risk and protective factors. Legend: Meta-regression analyses of all outcomes for which data were available from at least 20 studies. Results reflect the change in percentage points of knowledge of that risk factor across studies, adjusted (if applicable) for age, gender, education, and study population.

Meta-regression of the eight risk factors that were studied in at least 20 reports, showed that jointly, the included predictors explained 4.6%−57.3% of the variance in the models. No consistent study-level variation in risk factor knowledge by age, gender, educational attainment or quality appraisal was found (Fig. 4 and Supplemental Table S9). Studies among older populations tended to show slightly higher knowledge, but this was statistically significant only for excessive alcohol (β_age_=8.51, 95%CI=0.08–16.94). Compared to the general adult population, knowledge of hypertension and dyslipidaemia as risk factors was somewhat higher in studies among healthcare professionals (Fig. 4). Risk factor identification did not change significantly over time. In the adjusted models, knowledge of risk factors was generally highest in North America and Asia, notably for hypertension, dyslipidaemia, physical activity, and diet, with no notable differences between other continents. Results from high-quality articles only generally were similar to the overall estimates (Supplemental File S10). Funnel plots were somewhat asymmetrical for several risk factors, as indicated by a significant Egger’s test for hypertension (p = 0.01) and smoking (p = 0.03) (Supplemental File S11).

#### 3.3.2 Qualitative studies

Among the 23 qualitative studies on risk factor knowledge, ten (43.5%) used interviews, eight (34.8%) used focus group discussions and five (21.7%) a combination thereof. Almost all studies used a semi-structured topic guide, including dementia awareness (e.g. causes, symptoms, diagnostics), care and support services, perceived susceptibility, preventability, and attitudes towards prevention. Studies asked either about causes of dementia or about risk factors, generally not probing towards any risk factors in particular. Five studies used case vignettes of a person with dementia [[36], [37], [38], [39], [40]].

Non-modifiable factors like age, genetics, and family history came up in half of studies [[39], [40], [41], [42], [43], [44], [45], [46], [47], [48], [49], [50]]. In five articles it was reported that participants perceived cognitive impairment to be inevitable, that it could happen to anyone, and may be due to bad luck, suggesting little potential for modifiability of dementia risk [40,44,[51], [52], [53]]. In eighteen studies, participants identified modifiable risk factors, but participants were not unequivocally convinced about the causal contribution of these factors. The most-mentioned modifiable risk factors were cardiovascular and lifestyle factors (e.g., diet, exercise, smoking and alcohol consumption), but only in two studies participants implied the need for regular medical checks of cholesterol, blood pressure and diabetes in view of dementia risk reduction [39,41,44,45,47,48,[50], [51], [52],^54^,^55^]. Other frequently mentioned factors were psychosocial factors like loneliness, depression, stress, and cognitive stimulation, either as direct contributors to dementia risk or indirectly through other health conditions [37,40,41,[43], [44], [45], [46], [47], [48], [49], [50], [51], [52],^55^]. More ‘dementia‐specific’ risk factors such as head injury, hearing loss, and education were reported rarely [45,48,54]. Similarly, environmental factors like air pollution and pesticide exposure seldomly came up [44,45,54].

### 3.4 Dementia risk perception

#### 3.4.1 Quantitative studies

The constructs used to assess risk perception varied substantially between studies. Twenty-three of 50 studies (46.0%) measured risk perception directly, i.e., by asking for perceived risk of developing dementia later in life (‘perceived susceptibility’). Others gauged risk perception indirectly, in terms of fear of dementia (17/50, 34.0%), concern of developing dementia (12/50, 24.0%), worry about developing dementia (11/50, 22.0%) or perceived severity of having dementia (8/50, 16.0%). Of all 50 studies, 8 (16.0%) used a validated questionnaire to measure risk perception, notably the Dementia Worry Scale, items from the Motivation to Change Lifestyle and Health Behaviors for Dementia Risk Reduction Scale (MCHLB-DRR) – 4 statements on a scale from 5 (strongly disagree) to 20 (strongly agree), e.g., *‘My chances of developing dementia are high’*, or the Motivation to Change Behavior for Dementia Risk Reduction Scale (MOCHAD-10) [20,[56], [57], [58], [59], [60]]. Risk perception was predominantly measured on a 5-point Likert scale (34/50, 68.0%), while 7 (14.0%) studies used a visual analogue scale mostly ranging from 0 to 100, and 5 (10.0%) articles related to worry, concern and fear, reported yes/no responses to questions like *‘Do you worry about getting dementia?’* and *‘Do you have any fear of getting Alzheimer’s disease in later years?’.* Questions either included dementia in general (25/50, 50.0%), Alzheimer’s Disease (22/50, 44.0%), or both (3/50, 6.0%). Two articles compared individuals’ subjective risk with their objectively estimated risk, the latter determined using the Australian National University Alzheimer's Disease Risk Index, or gender- and genotype-specific Alzheimer’s disease risk [61,62].

Due to the variety in assessment methods, we could not quantitatively synthesize findings across all articles. Still, 6/23 studies that used the perceived susceptibility subscale of the MCHLB-DRR were pooled (range 5–20), resulting in a median perceived susceptibility of 10.8 (IQR: 9.4–11.3) [[57], [58], [59], [60],^63^,^64^]. Three studies that assessed perceived lifetime risk of Alzheimer’s disease reported a median of 52.3% (IQR: 50.3–54.2%) [61,62,65]. In one of these studies, comparing subjective to objective risk, 87.5% of older adults overestimated their personal risk by at least 10% [61]. In another study among older adults, 56.3% estimated their own risk to be lower than that of a typical person their age and gender, whereas only 8.5% estimated their risk to be higher [66]. With regard to levels of worry, concern and fear of dementia, variation across articles was substantial. For instance, the mean percentage of participants that worries about developing dementia ranged from 29.4 to 86.5% [[67], [68], [69], [70], [71], [72]].

Among the few studies that compared risk perception across setting or population, perceived risk was somewhat higher among individuals with 2 or more relatives with Alzheimer’s disease [65]. Perceived risk was also higher among individuals with mild cognitive impairment than in cognitively healthy individuals [73]. In a single study on gender differences, women reported higher levels of perceived susceptibility, worry and fear about Alzheimer’s disease than men [71].

#### 3.4.2 Qualitative studies

In four qualitative studies on risk perception, the majority of adults expressed a fear towards developing dementia, either for themselves or for loved ones [44,45,74,75]. Several participants indicated dementia as one of their most feared health threats, more than any other disease. Reasons for this related to the disease itself (e.g., loss of control and disease insight), its impact on relationships, and stigma around the disease.

## 4 Discussion

In this systematic review and meta-analysis of data from 164,644 individuals across 41 countries, knowledge of dementia risk and protective factors remains limited, particularly regarding cardiovascular and environmental risk factors. Of the 14 established modifiable dementia risk factors, 11 were recognised by fewer than half of individuals, without clear demographic subgroup differences. Articles on direct risk perception demonstrated a moderate risk perception, with large variation across studies, while the qualitative studies generally highlighted participants expressing a fear towards developing dementia.

Of the risk and protective factors acknowledged by the Lancet Commission on Dementia Prevention, Intervention and Care, physical activity, traumatic brain injury and social isolation were correctly identified by two thirds of participants, whereas risk factor recognition was particularly poor for obesity, air pollution, and educational attainment [5]. People generally seemed more convinced about factors for which evidence is relatively weak (e.g., low cognitive activity, stress) than about the more robustly established risk factors like hypertension. Both quantitative and qualitative studies reported low levels of knowledge of environmental risk factors and education. The scientific evidence supporting a causal role of these risk factors in dementia has increased in recent years, underlining the need to bring public awareness on par [5,[76], [77], [78]]. This applies to risk factors that are amendable on an individual level, as well as those relying mostly on public health policy interventions to sort effect (e.g., air pollution). In addition, as the field of dementia prevention is rapidly developing, novel modifiable risk factors are emerging, highlighting the importance of updated education strategies and recurrent assessment of awareness [[79], [80], [81], [82]]. Of note, most studies included in this review assessed risk factor recognition, rather than recall, which may overestimate the degree to which knowledge is readily available to people in their everyday life [83,84]. Assessment of recall, rather than recognition, and integration of decoy answers into standardised tools like the ADKS could facilitate valid assessment of risk factor knowledge. These tools need to be revised periodically to reflect the evolving scientific evidence around dementia risk factors [84,85].

Although risk factor awareness was for the most part limited, the substantial heterogeneity in the presented results calls for a careful interpretation. Differences between studies may have arisen from methodological differences or actual population variability. Based on the meta-regression results, we could not pinpoint the precise causes of this heterogeneity, and there were no easily identifiable demographic subgroups in which knowledge is much lower or higher than in others. Whilst earlier systematic reviews reported that higher education was correlated with better risk factor knowledge, this signal is no longer clear in this review including additional studies from the last five years [25,27]. It should be noted that the share of people with lower education across studies was relatively limited. Older individuals encounter more peers with dementia, and generally have a higher risk perception, but in the current review absolute differences in risk factor knowledge with age were relatively small [27,65,86,87]. The absence of consistent time trends in risk factor knowledge provide no support to presumed increases in overall dementia risk factor knowledge due to increased awareness and public education. However, such intervention effects are best mapped in direct before-after designs, which has provided some evidence of moderate campaign efficacy in the past [21,23]. Taken together, these results suggest that public education campaigns are best targeted broadly, although communication style and content may be tailored to the demographic subgroups predominantly represented in various media outlets.

Dementia risk perception varied greatly across studies, and heterogeneity in assessment of risk perception hampered overarching conclusions. On a study level, risk perception was generally around the median of the scale (e.g., 10.8/20 and 52.3/100), indicating a moderate risk. On the basis of research in oncology, level of risk perception depends heavily on the type of measure used [88,89]./ Numeric estimates (e.g., 0 to 100) often were overestimations, while comparative measures (e.g., comparing one’s own risk to that of others) were more accurate. A combination of both may be preferable to promote accuracy. The qualitative studies in our review emphasised participants’ concern and anxiety about developing dementia. This fear may be amplified by the misconception that dementia is an inevitable part of aging, or that it is solely determined by family history [90,91]. Such deterministic views may discourage people from adopting preventive health behaviours. While anxiety and fear can be distressing, it also presents an opportunity; when properly harnessed, they can be transformed into greater knowledge and positive attitudes toward prevention.

The limited knowledge of dementia risk and protective factors, and the lack of clear improvement over the past two decades, underscore the urgent need for educational initiatives [17]. For cardiometabolic lifestyle factors, like obesity or smoking, we believe this would best be integrated into prevention initiatives against for example cardiovascular disease and cancer [92]. Dementia-specific risk factors like air pollution and hearing impairment may warrant a more tailored approach. When designing such interventions, the socio-economic context of a country or region should be considered, e.g., an integration of dementia prevention strategies into existing initiatives may be particularly worthwhile in low-resource environments. Regional population-attributable fractions for dementia could further guide prioritisation of education as well as preventive intervention strategies.

Stronger beliefs about dementia preventability could endorse preventive behaviours [93]. While people tend to become more knowledgeable through educational material, and knowledge leads to a greater *intention* to adopt healthier behaviours, actual lifestyle change often fails to materialise [17,[19], [20], [21],[94], [95], [96]]. This disconnect may occur particularly when perceived risk is low, like for dementia in adolescence or midlife [97]. Notwithstanding the importance of raising public health awareness of dementia risk factors and modifiability, optimal prevention in these groups may not be attainable without a broader, population-level approach that reduces overall risk factor exposure. Such approaches could promote a healthy society through for example green space, air quality regulation, and taxation of tobacco and unhealthy food [13,98]. The combination of population-level and individual-level educational interventions would also effectively reach the –on the basis of this review– overwhelming share of the population that is currently unaware of the dementia risk factors, or unable to effectively modify their health through lifestyle change.

Strengths of this study include the comprehensive literature search, and aggregation of both quantitative and qualitative data, regardless of geographic location or population. This allowed the most comprehensive overview of public awareness of dementia risk to date, including meta-regression analyses to identify subgroups of the population for which outcomes differ. Several limitations also need to be accounted for. First, restriction to the English language and exclusion of grey literature may have hampered geographical dispersity, even though countries from all continents were represented in this review. As certain countries, such as the USA and China, were highly represented, levels of knowledge per continent may not be representative for other, especially lower-income countries. Second, reference lists of included articles and reviews were not scrutinized for additional relevant studies, and therefore some studies may have been missed. Third, we combined knowledge of factors framed as a risk or as protective (e.g., too much sleep and too little sleep were aggregated to “sleep”), which may have impacted knowledge levels. Fourth, most studies assessed risk factor recognition rather than recall, which may not reflect how decisions are informed in everyday life. Fifth, some asymmetry in the funnel plots may be due either to underreporting or methodological heterogeneity; as the descriptives study in this review generally do not test a certain hypothesis, we believe publication bias to be less likely. Finally, limitations in the sampling method and response rate of the included studies potentially hampers generalizability and may have caused sampling bias, as potentially particularly individuals with an interest in research, and dementia prevention specifically, have been included.

## 5 Conclusion

Knowledge of risk and protective factors for dementia in the general population remains limited. The lack of clear evolution in risk factor knowledge over the past 15 years calls for a combination of public education to promote awareness, as well as population-level initiatives that reduce risk factor burdens on a societal level. Monitoring the effects of public education on knowledge and actual behavioural change is essential, and could benefit from meticulous and standardized assessment methods that include active recall to most likely reflect knowledge availability in every-day life.

## Ethical statement

• Studies on Human and/or Animal statement: Not applicable.
• Informed consent: Not applicable.
• Approval ± registration number: CRD420250636873

## Declaration of the use of generative AI and AI-assisted technologies in scientific writing and in figures, images and artwork

We used Claude, version 2025, only for a more concise rewriting of a couple sentences in the discussion section of the manuscript, which did not affect the formulation of the conclusions and did not require sharing of any research data with the algorithm.

## Funding

This work is part of the BIRD-NL consortium funded by the Netherlands Organisation for Health Research and Development (ZonMw) as part of the National Dementia Strategy 2021–2030 by the Dutch Ministry of Health, Welfare and Sport (grant number: 1051003210005)

## Data statement

Data can be made available upon reasonable request from the corresponding author (f.j.wolters@erasmusmc.nl)

## CRediT authorship contribution statement

**Muhammed L. Sambou:** Writing – review & editing, Writing – original draft, Methodology, Formal analysis, Data curation. **Jolanda H.M. Dobbe:** Writing – review & editing, Writing – original draft, Methodology, Formal analysis, Data curation. **Elaine A.C. Albers:** Writing – review & editing, Investigation. **Kay Deckers:** Writing – review & editing. **Lidia R. Arends:** Writing – review & editing. **M. Arfan Ikram:** Writing – review & editing, Supervision, Conceptualization. **Ellen M.A. Smets:** Writing – review & editing, Conceptualization. **Wichor M. Bramer:** Writing – review & editing, Data curation. **Jeremy A. Labrecque:** Writing – review & editing, Supervision. **Leonie N.C. Visser:** Writing – review & editing, Supervision, Conceptualization. **Frank J. Wolters:** Writing – review & editing, Supervision, Funding acquisition, Conceptualization.

## Declaration of competing interest

None declared.

## Appendix Supplementary materials

Image, application 1
