# Supplementary material for: Knowledge and perception of dementia risk and protective factors: a systematic review and meta-analysis
Source: J Prev Alzheimers Dis. 2026 Apr 11;13(6):100565. doi: 10.1016/j.tjpad.2026.100565 (PMC13092043; doi:10.1016/j.tjpad.2026.100565)
Supplement: Supplementary file 1 [file mmc1.docx]

**Online supplementary material**

**Knowledge and perception of dementia risk and protective factors: a systematic review and meta-analysis**

Muhammed L. Sambou^1†^, Jolanda H.M. Dobbe^2,3,4,5†^, Elaine A.C. Albers^2^, Kay Deckers^6^, Lidia R. Arends^7^, M. Arfan Ikram^1^, Ellen M.A. Smets^2,4,8^, Wichor M. Bramer^9^, Jeremy A. Labrecque^1^, Leonie N.C. Visser ^2,4,8,10,11^, Frank J. Wolters^1,12^

^1^ Department of Epidemiology, Erasmus MC University Medical Center, Rotterdam, The Netherlands

^2^ Department of Medical Psychology, Amsterdam University Medical Center location AMC, University of Amsterdam, Amsterdam, The Netherlands

^3^ Alzheimer Center Amsterdam, Neurology, Vrije Universiteit Amsterdam, Amsterdam UMC location VUmc, Amsterdam, The Netherlands.

^4^ Amsterdam Public Health, Quality of Care, Amsterdam, The Netherlands

^5^ Amsterdam Public Health, Ageing and later life, Amsterdam, The Netherlands

^6^ Alzheimer Centre Limburg, Department of Psychiatry and Neuropsychology, Mental Health and Neuroscience Research Institute (MHeNs), Maastricht University, Maastricht, The Netherlands

^7^ Department of Biostatistics, Erasmus Medical Center, Rotterdam, The Netherlands

^8^ Amsterdam Public Health, Personalized medicine, Amsterdam, The Netherlands

^9^ Medical Library, Erasmus MC University Medical Center, Rotterdam, The Netherlands

^10^ Division of Clinical Geriatrics, Center for Alzheimer Research, Department of Neurobiology, Care Sciences and Society, Karolinska Institutet, Stockholm, Sweden

^11^ Department of Bioethics and Health Humanities, Julius Center for Health Sciences and Primary Care, University Medical Center Utrecht, Utrecht University, Utrecht, The Netherlands

^12^ Department of Radiology & Nuclear Medicine and Alzheimer Center, Erasmus MC University Medical Center, Rotterdam, The Netherlands

† These authors contributed equally to this work

Corresponding author:

Frank J. Wolters, MD PhD

Dr. Molewaterplein 40, PO Box 2040, 3000 CA Rotterdam

Email: [f.j.wolters@erasmusmc.nl](mailto:f.j.wolters@erasmusmc.nl)

Phone: +31 107 034 625

**Supplemental File S1: Search strategy**

**Supplemental File S2: Formula to approximate the median age**

**Supplemental File S3: Critical Appraisal Checklist for studies reporting prevalence data**

**Supplemental File S4: Quality assessment of quantitative papers**

**Supplemental File S5: JBI Critical Appraisal Checklist for Qualitative Research**

**Supplemental File S6: Quality assessment of qualitative papers**

**Supplemental File S7: Knowledge of risk and protective factors of dementia**

**Supplemental File S8: Comparison of recall and recognition of risk factors in the general population**

**Supplemental File S9: Meta-regression analysis of all risk and protective factors assessed in at least 20 studies**

**Supplemental File S10: Secondary analysis of risk factor knowledge with high-quality articles**

**Supplemental File S11: Funnel plots and Egger’s regression test of all risk and protective factors included in meta-regression**

**Supplemental File S1: Search strategy**

| **Database searched** | **Platform** | **Years of coverage** | **Records** | **Records after duplicates removed** |
| --- | --- | --- | --- | --- |
| Medline ALL | Ovid | 1946 - Present | 1593 | 1584 |
| Embase | Embase.com | 1971 - Present | 3024 | 1681 |
| Web of Science Core Collection* | Web of Knowledge | 1975 - Present | 2840 | 1397 |
| Cochrane Central Register of Controlled Trials** | Wiley | 1992 - Present | 91 | 51 |
| PsycINFO | Ovid | 1806 - Present | 951 | 286 |
| **Total** | | | **8499** | **4999** |

*Science Citation Index Expanded (1975-present) ; Social Sciences Citation Index (1975-present) ; Arts & Humanities Citation Index (1975-present) ; Conference Proceedings Citation Index- Science (1990-present) ; Conference Proceedings Citation Index- Social Science & Humanities (1990-present) ; Emerging Sources Citation Index (2005-present)

** Manually deleted abstracts from trial registries

No other database limits were used than those specified in the search strategies

**Medline 1593**

(* Awareness / OR * Knowledge / OR * Perception / OR Public Opinion / OR * Attitude to Health / OR (awareness OR knowledge* OR misunderstand* OR belief* OR perception* OR opinion OR attitude* OR know OR misconception* OR ignorance*).ti. OR ((awareness OR knowledge* OR misunderstand* OR belief* OR perception* OR opinion* OR attitude* OR know OR misconception* OR ignorance*) ADJ6 (general* OR population* OR patient* OR public* OR community OR lay OR laymen* OR layperson* OR individual*)).ab,ti,kw.) AND (exp Dementia/ OR (dementia* OR alzheimer*).ab,ti,kw.) AND (Risk Factors/ OR Risk Reduction Behavior/ OR * Risk/ OR Exercise/ OR exp Diet/ OR Antioxidants/ OR exp Eating/ OR exp Diabetes Mellitus/ OR exp Overnutrition/ OR Body Mass Index/ OR Anthropometry / OR exp Body Weights and Measures / OR exp Hyperlipidemias/ OR exp Hypertension / OR exp Smoking / OR exp Smoking Devices/ OR exp Substance-Related Disorders/ OR exp Educational Status/ OR exp Socioeconomic Factors/ OR pollution/ OR Pesticides/ OR Endotoxins/ OR exp Depression/ OR exp Depressive Disorder/ OR Mental Disorders/ OR Psychological Trauma / OR exp Hearing Loss/ OR exp Vision Disorders/ OR Brain Injuries, Traumatic/ OR exp Craniocerebral Trauma/ OR exp Social Behavior/ OR exp Heart Diseases/ OR exp Renal Insufficiency/ OR exp Sleep / OR Stress, Psychological/ OR exp Medical History Taking/ OR Mutation/ OR Genotype/ OR Heredity/ OR exp Genetics/ OR Parks, Recreational/ OR exp Residence Characteristics/ OR exp Substance-Related Disorders/ OR exp Sports/ OR exp Athletes/ OR exp Medication Adherence/ OR (((risk OR protecti* OR prevent*) ADJ3 (factor* OR reduction*)) OR ((physical* OR cognitive* OR mental*) ADJ3 (activ* OR inactiv*)) OR sedentary OR diet* OR antioxidant* OR ((fat OR alcohol*) ADJ3 (intake* OR consumption* OR "use" OR abuse)) OR ketogenic* OR diabet* OR overnutrition* OR overweight* OR obes* OR body-mass* OR bmi OR anthropometr* OR ((cholesterol* OR lipid*) ADJ3 (blood OR level* OR high OR elevat* OR disorder*)) OR hypercholesterol* OR hyperlipid* OR blood-pressure* OR Hypertensi* OR smoking OR cigarette* OR socioeconomic* OR socio-economic* OR ((education*) ADJ3 (status OR low* OR level*)) OR pollution* OR pesticide* OR endotoxin* OR depressi* OR ((mental* OR psychiatr* OR psycholog*) ADJ3 (ill* OR disease* OR disorder*)) OR psychotraum* OR (emotion* ADJ3 trauma*) OR ((hearing OR visual) ADJ3 (impairment* OR loss)) OR deaf* OR blind* OR ((trauma* OR injur*) ADJ3 (brain* OR head)) OR (social ADJ3 (behav* OR isolat* OR activit*)) OR ((heart OR cardiac* OR Coronar*) ADJ3 (disease*)) OR ((kidney* OR renal) ADJ3 (failure* OR disease* OR chronic* OR poor)) OR (sleep* ADJ3 (parameter* OR qualit* OR pattern* OR disturb*)) OR bedtime* OR bed-time* OR insomn* OR narcolop* OR chronotype* OR stress OR ((parent* OR family) ADJ3 dementia) OR (famil* ADJ3 history) OR ancestr* OR mutation* OR genotype* OR heredit* OR genetic* OR ((green* OR public) ADJ3 space*) OR ((residen* OR neighbourhood) ADJ3 characteristic*) OR sport* OR athlet* OR ((medication* OR drug*) ADJ3 (complian* OR adher* OR noncomplian* OR nonadher*)) OR demograph*).ab,ti,kw. OR (risk* OR prevention*).ti.) AND 2010:2024.(sa_year). NOT ((exp * Attitude of Health Personnel / OR exp * Health Personnel / OR (nurse* OR nursing OR staff OR personnel* OR professional* OR doctor* OR physician* OR provider*).ti.) NOT (* Patients / OR (patient* OR general* OR public*).ti.))

**Embase 3024**

(awareness/mj OR knowledge/mj OR perception/mj OR 'public opinion'/mj OR 'attitude to health'/mj OR 'patient attitude'/mj OR (awareness OR knowledge* OR understand* OR misunderstand* OR belief* OR perception* OR opinion OR attitude* OR know OR misconception* OR ignorance*):ti OR ((awareness OR knowledge* OR understand* OR misunderstand* OR belief* OR perception* OR opinion* OR attitude* OR know OR misconception* OR ignorance*) NEAR/6 (general* OR population* OR patient* OR public* OR community OR lay OR laymen* OR layperson* OR individual*)):ab,ti,kw) AND (dementia/exp OR (dementia* OR alzheimer*):Ab,ti,kw) AND ('risk factor'/de OR 'risk reduction'/de OR protection/de OR risk/mj OR 'physical activity'/exp OR diet/exp OR antioxidant/de OR 'dietary intake'/exp OR 'diabetes mellitus'/exp OR 'diabetic patient'/de OR overnutrition/exp OR 'body mass'/de OR 'anthropometric parameters'/exp OR 'lipid blood level'/de OR hyperlipidemia/exp OR 'abnormal blood pressure'/exp OR 'smoking and smoking related phenomena'/exp OR 'smoking device'/exp OR 'substance use'/exp OR 'educational status'/exp OR socioeconomics/de OR pollution/exp OR pesticide/exp OR endotoxin/de OR depression/exp OR 'mental disease'/de OR 'psychological aspect'/de OR psychotrauma/de OR 'hearing impairment'/exp OR 'visual impairment'/exp OR 'traumatic brain injury'/exp OR 'head injury'/exp OR 'social behavior'/exp OR 'heart disease'/exp OR 'kidney failure'/exp OR 'sleep parameters'/exp OR 'mental stress'/exp OR 'family history'/de OR mutation/exp OR genotype/de OR heredity/de OR genetics/de OR 'green space'/de OR 'residence characteristics'/de OR neighborhood/de OR 'drug abuse'/exp OR sport/exp OR athlete/exp OR 'medication compliance'/exp OR (((risk OR protecti* OR prevent*) NEAR/3 (factor* OR reduction*)) OR ((physical* OR cognitive* OR mental*) NEAR/3 (activ* OR inactiv*)) OR sedentary OR diet* OR antioxidant* OR ((fat OR alcohol*) NEAR/3 (intake* OR consumption* OR use OR abuse)) OR ketogenic* OR diabet* OR overnutrition* OR overweight* OR obes* OR body-mass* OR bmi OR anthropometr* OR ((cholesterol* OR lipid*) NEAR/3 (blood OR level* OR high OR elevat* OR disorder*)) OR hypercholesterol* OR hyperlipid* OR blood-pressure* OR Hypertensi* OR smoking OR cigarette* OR socioeconomic* OR socio-economic* OR ((education*) NEAR/3 (status OR low* OR level*)) OR pollution* OR pesticide* OR endotoxin* OR depressi* OR ((mental* OR psychiatr* OR psycholog*) NEAR/3 (ill* OR disease* OR disorder*)) OR psychotraum* OR (emotion* NEAR/3 trauma*) OR ((hearing OR visual) NEAR/3 (impairment* OR loss)) OR deaf* OR blind* OR ((trauma* OR injur*) NEAR/3 (brain* OR head)) OR (social NEAR/3 (behav* OR isolat* OR activit*)) OR ((heart OR cardiac* OR Coronar*) NEAR/3 (disease*)) OR ((kidney* OR renal) NEAR/3 (failure* OR disease* OR chronic* OR poor)) OR (sleep* NEAR/3 (parameter* OR qualit* OR pattern* OR disturb*)) OR bedtime* OR bed-time* OR insomn* OR narcolop* OR chronotype* OR stress OR ((parent* OR family) NEAR/3 dementia) OR (famil* NEAR/3 history) OR ancestr* OR mutation* OR genotype* OR heredit* OR genetic* OR ((green* OR public) NEAR/3 space*) OR ((residen* OR neighbourhood) NEAR/3 characteristic*) OR sport* OR athlet* OR ((medication* OR drug*) NEAR/3 (complian* OR adher* OR noncomplian* OR nonadher*)) OR demograph*):ab,ti,kw OR (risk* OR prevention*):ti) NOT [conference abstract]/lim AND [2010-2024]/py NOT (('health personnel attitude'/exp/mj OR 'health care personnel'/exp/mj OR (nurse* OR nursing OR staff OR personnel* OR professional* OR doctor* OR physician* OR provider*):ti) NOT ('patient attitude'/exp/mj OR patient/exp/mj OR (patient* OR general* OR public*):ti))

**Web of science 2840**

(TI=(awareness OR knowledge* OR misunderstand* OR belief* OR perception* OR opinion OR attitude* OR know OR misconception* OR ignorance*) OR TS=((awareness OR knowledge* OR misunderstand* OR belief* OR perception* OR opinion* OR attitude* OR know OR misconception* OR ignorance*) NEAR/5 (general* OR population* OR patient* OR public* OR community OR lay OR laymen* OR layperson* OR individual*))) AND TS=((dementia* OR alzheimer*)) AND (TS=(((risk OR protecti* OR prevent*) NEAR/2 (factor* OR reduction*)) OR ((physical* OR cognitive* OR mental*) NEAR/2 (activ* OR inactiv*)) OR sedentary OR diet* OR antioxidant* OR ((fat OR alcohol*) NEAR/2 (intake* OR consumption* OR use OR abuse)) OR ketogenic* OR diabet* OR overnutrition* OR overweight* OR obes* OR body-mass* OR bmi OR anthropometr* OR ((cholesterol* OR lipid*) NEAR/2 (blood OR level* OR high OR elevat* OR disorder*)) OR hypercholesterol* OR hyperlipid* OR blood-pressure* OR Hypertensi* OR smoking OR cigarette* OR socioeconomic* OR socio-economic* OR ((education*) NEAR/2 (status OR low* OR level*)) OR pollution* OR pesticide* OR endotoxin* OR depressi* OR ((mental* OR psychiatr* OR psycholog*) NEAR/2 (ill* OR disease* OR disorder*)) OR psychotraum* OR (emotion* NEAR/2 trauma*) OR ((hearing OR visual) NEAR/2 (impairment* OR loss)) OR deaf* OR blind* OR ((trauma* OR injur*) NEAR/2 (brain* OR head)) OR (social NEAR/2 (behav* OR isolat* OR activit*)) OR ((heart OR cardiac* OR Coronar*) NEAR/2 (disease*)) OR ((kidney* OR renal) NEAR/2 (failure* OR disease* OR chronic* OR poor)) OR (sleep* NEAR/2 (parameter* OR qualit* OR pattern* OR disturb*)) OR bedtime* OR bed-time* OR insomn* OR narcolop* OR chronotype* OR stress OR ((parent* OR family) NEAR/2 dementia) OR (famil* NEAR/2 history) OR ancestr* OR mutation* OR genotype* OR heredit* OR genetic* OR ((green* OR public) NEAR/2 space*) OR ((residen* OR neighbourhood) NEAR/2 characteristic*) OR sport* OR athlet* OR ((medication* OR drug*) NEAR/2 (complian* OR adher* OR noncomplian* OR nonadher*)) OR demograph*) OR TI=(risk* OR prevention*)) AND PY=(2010-2024) NOT DT=(Meeting Abstract OR Meeting Summary) NOT TI=(((nurse* OR nursing OR staff OR personnel* OR professional* OR doctor* OR physician* OR provider*)) NOT ((patient* OR general* OR public*)))

**Cochrane 91**

((awareness OR knowledge* OR misunderstand* OR belief* OR perception* OR opinion OR attitude* OR know OR misconception* OR ignorance*):ti OR ((awareness OR knowledge* OR misunderstand* OR belief* OR perception* OR opinion* OR attitude* OR know OR misconception* OR ignorance*) NEAR/6 (general* OR population* OR patient* OR public* OR community OR lay OR laymen* OR layperson* OR individual*)):ab,ti,kw) AND ((dementia* OR alzheimer*):Ab,ti,kw) AND ((((risk OR protecti* OR prevent*) NEAR/3 (factor* OR reduction*)) OR ((physical* OR cognitive* OR mental*) NEAR/3 (activ* OR inactiv*)) OR sedentary OR diet* OR antioxidant* OR ((fat OR alcohol*) NEAR/3 (intake* OR consumption* OR use OR abuse)) OR ketogenic* OR diabet* OR overnutrition* OR overweight* OR obes* OR body-mass* OR bmi OR anthropometr* OR ((cholesterol* OR lipid*) NEAR/3 (blood OR level* OR high OR elevat* OR disorder*)) OR hypercholesterol* OR hyperlipid* OR blood-pressure* OR Hypertensi* OR smoking OR cigarette* OR socioeconomic* OR socio-economic* OR ((education*) NEAR/3 (status OR low* OR level*)) OR pollution* OR pesticide* OR endotoxin* OR depressi* OR ((mental* OR psychiatr* OR psycholog*) NEAR/3 (ill* OR disease* OR disorder*)) OR psychotraum* OR (emotion* NEAR/3 trauma*) OR ((hearing OR visual) NEAR/3 (impairment* OR loss)) OR deaf* OR blind* OR ((trauma* OR injur*) NEAR/3 (brain* OR head)) OR (social NEAR/3 (behav* OR isolat* OR activit*)) OR ((heart OR cardiac* OR Coronar*) NEAR/3 (disease*)) OR ((kidney* OR renal) NEAR/3 (failure* OR disease* OR chronic* OR poor)) OR (sleep* NEAR/3 (parameter* OR qualit* OR pattern* OR disturb*)) OR bedtime* OR bed-time* OR insomn* OR narcolop* OR chronotype* OR stress OR ((parent* OR family) NEAR/3 dementia) OR (famil* NEAR/3 history) OR ancestr* OR mutation* OR genotype* OR heredit* OR genetic* OR ((green* OR public) NEAR/3 space*) OR ((residen* OR neighbourhood) NEAR/3 characteristic*) OR sport* OR athlet* OR ((medication* OR drug*) NEAR/3 (complian* OR adher* OR noncomplian* OR nonadher*)) OR demograph*):ab,ti,kw OR (risk* OR prevention*):ti)

**PsycINFO 951**

(* Awareness / OR * Knowledge / OR * Perception / OR Public Opinion / OR * Attitude to Health / OR (awareness OR knowledge* OR misunderstand* OR belief* OR perception* OR opinion OR attitude* OR know OR misconception* OR ignorance*).ti. OR ((awareness OR knowledge* OR misunderstand* OR belief* OR perception* OR opinion* OR attitude* OR know OR misconception* OR ignorance*) ADJ6 (general* OR population* OR patient* OR public* OR community OR lay OR laymen* OR layperson* OR individual*)).ab,ti.) AND (exp Dementia/ OR (dementia* OR alzheimer*).ab,ti.) AND (Risk Factors/ OR Risk Reduction Behavior/ OR * Risk/ OR Exercise/ OR exp Diet/ OR Antioxidants/ OR exp Eating/ OR exp Diabetes Mellitus/ OR exp Overnutrition/ OR Body Mass Index/ OR Anthropometry / OR exp Body Weights and Measures / OR exp Hyperlipidemias/ OR exp Hypertension / OR exp Smoking / OR exp Smoking Devices/ OR exp Substance-Related Disorders/ OR exp Educational Status/ OR exp Socioeconomic Factors/ OR pollution/ OR Pesticides/ OR Endotoxins/ OR exp Depression/ OR exp Depressive Disorder/ OR Mental Disorders/ OR Psychological Trauma / OR exp Hearing Loss/ OR exp Vision Disorders/ OR Brain Injuries, Traumatic/ OR exp Craniocerebral Trauma/ OR exp Social Behavior/ OR exp Heart Diseases/ OR exp Renal Insufficiency/ OR exp Sleep / OR Stress, Psychological/ OR exp Medical History Taking/ OR Mutation/ OR Genotype/ OR Heredity/ OR exp Genetics/ OR Parks, Recreational/ OR exp Residence Characteristics/ OR exp Substance-Related Disorders/ OR exp Sports/ OR exp Athletes/ OR exp Medication Adherence/ OR (((risk OR protecti* OR prevent*) ADJ3 (factor* OR reduction*)) OR ((physical* OR cognitive* OR mental*) ADJ3 (activ* OR inactiv*)) OR sedentary OR diet* OR antioxidant* OR ((fat OR alcohol*) ADJ3 (intake* OR consumption* OR "use" OR abuse)) OR ketogenic* OR diabet* OR overnutrition* OR overweight* OR obes* OR body-mass* OR bmi OR anthropometr* OR ((cholesterol* OR lipid*) ADJ3 (blood OR level* OR high OR elevat* OR disorder*)) OR hypercholesterol* OR hyperlipid* OR blood-pressure* OR Hypertensi* OR smoking OR cigarette* OR socioeconomic* OR socio-economic* OR ((education*) ADJ3 (status OR low* OR level*)) OR pollution* OR pesticide* OR endotoxin* OR depressi* OR ((mental* OR psychiatr* OR psycholog*) ADJ3 (ill* OR disease* OR disorder*)) OR psychotraum* OR (emotion* ADJ3 trauma*) OR ((hearing OR visual) ADJ3 (impairment* OR loss)) OR deaf* OR blind* OR ((trauma* OR injur*) ADJ3 (brain* OR head)) OR (social ADJ3 (behav* OR isolat* OR activit*)) OR ((heart OR cardiac* OR Coronar*) ADJ3 (disease*)) OR ((kidney* OR renal) ADJ3 (failure* OR disease* OR chronic* OR poor)) OR (sleep* ADJ3 (parameter* OR qualit* OR pattern* OR disturb*)) OR bedtime* OR bed-time* OR insomn* OR narcolop* OR chronotype* OR stress OR ((parent* OR family) ADJ3 dementia) OR (famil* ADJ3 history) OR ancestr* OR mutation* OR genotype* OR heredit* OR genetic* OR ((green* OR public) ADJ3 space*) OR ((residen* OR neighbourhood) ADJ3 characteristic*) OR sport* OR athlet* OR ((medication* OR drug*) ADJ3 (complian* OR adher* OR noncomplian* OR nonadher*)) OR demograph*).ab,ti. OR (risk* OR prevention*).ti.) AND 2010:2024.(sa_year). NOT ((exp * Attitude of Health Personnel / OR exp * Health Personnel / OR (nurse* OR nursing OR staff OR personnel* OR professional* OR doctor* OR physician* OR provider*).ti.) NOT (* Patients / OR (patient* OR general* OR public*).ti.))

**Supplemental File S2: Formula to approximate the median age**

Formula

Median age = LL + $\frac{50\%-\sum\%all lower age categories}{( \frac{\% within median age category}{class width} )}$, where

- LL = the lower limit of the age category that includes the median age value of the overall study population
- ∑ all lower age categories = the cumulative % of participants included in all age categories below the category that includes the median age value of the overall study population
- % within median age category = the percentage of participants in the age category that includes the median age value of the overall study population
- Class width = the number of years in the age category that includes the median age value of the overall study population

Example:

Study: Imre, N. et al., 2019

Data:

25–35 years: 5.9%

36–45 years: 12.5%

46–55 years: 24.9%

56–65 years: 40.2%

>65 years: 16.5%

Approximate median age = 56 + $\frac{50-(5.9+12.5+24.9)}{\frac{40.2}{10}}$ = 57.7 years

**Supplemental File S3: Critical Appraisal Checklist for studies reporting prevalence data.^31^**


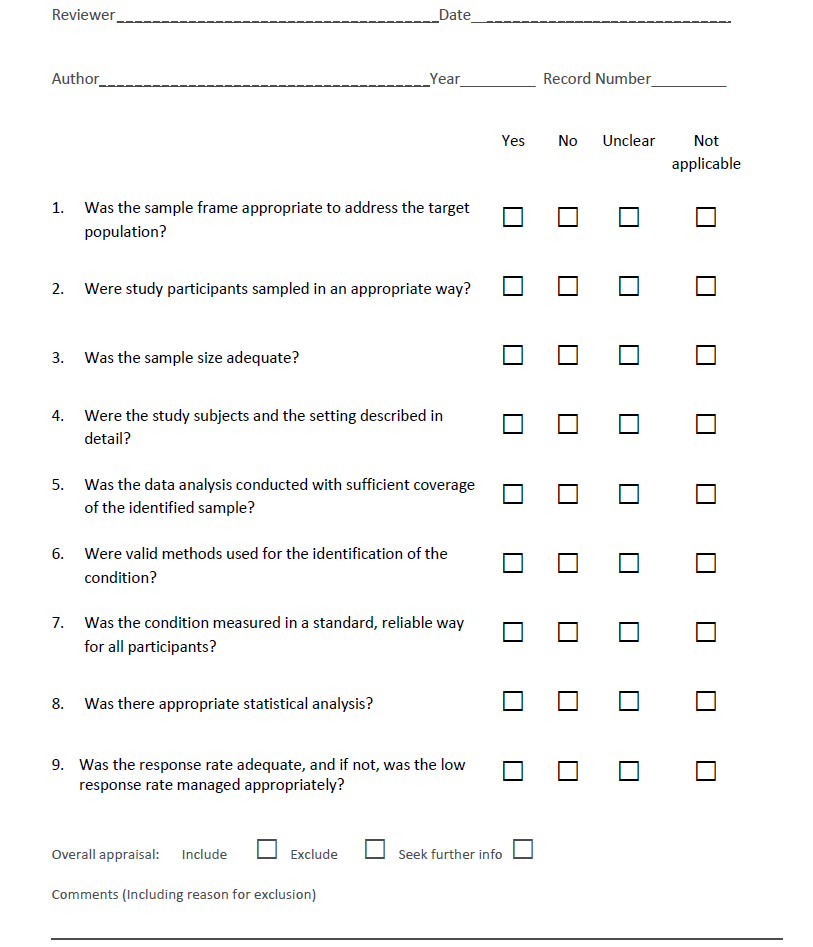


Notes.

Q1: Yes if sample characteristics such as age range and gender are reported and this aligns with the research question.

Q2: Yes if probability sampling was used; no if non-probability sampling was used; unclear if the sampling method was not (clearly) described.

Q3: Yes if sample size > 50, and described how this sample size was calculated. In case the sample is very large, a sample size calculation is not needed and the sample size can be considered as adequate.

Q4: Yes if methods section is elaborative enough to be able to redo the study. Especially the description of which participants are included and how they are included.

Q5: Yes, if for example, there is a good distribution between men and women, all age and education categories are well covered etc.

Q6: Yes if the questionnaires used are validated, or if the questionnaire is based on validated questionnaires and the questions are copied to their own questionnaire. No if the questionnaire is based on validated questionnaires, but the questions are adapted and/or only a small selection of questions is used. Also, if not described how these questionnaires were used (for example they mention that they based their questions on earlier questionnaires but not which questionnaires / what specific questions (and if they adapted these questions), this question was answered with ‘no’/’unclear’.

Q7: Yes if assessment has been performed in a consistent way. For example all questionnaires were self-administered. In case of interview-assisted questionnaires, check whether interviewers were trained in the use of the instrument. If more than 1 data collector, check if they were the same in terms of level of education, experience, and responsibility in the study.

Q8: Yes if statistical analyses were described clearly, making it possible to reproduce the analyses. And the analyses seem appropriate for answering the research question.

Q9: Yes if response rate is known or could be calculated based on the amount of people invited in the study, and the amount of included participants. If the response rate is low, but they explain what were reasons for not-responding/ whether responders and non-responders differed in characteristics, it could still be considered as yes. Unclear, if response rate is not known or could not be calculated. No, if response late is <50% and no information is provided on how they handled the low response rate.

**Supplemental File S4: Quality assessment of quantitative papers.**

| Author | Q1 | Q2 | Q3 | Q4 | Q5 | Q6 | Q7 | Q8 | Q9 | Overall appraisal |
| --- | --- | --- | --- | --- | --- | --- | --- | --- | --- | --- |
| Ahuja, R. et al., 2018 | Yes | No | Yes | Yes | Yes | No | Yes | Yes | Yes | 7 |
| Aihara, Y. and Maeda, K. 2020 | Yes | No | Yes | Yes | Yes | Yes | Yes | Yes | Yes | 8 |
| Aihara, Y. et al., 2020 | Yes | No | Yes | Yes | No | No | Yes | Yes | Yes | 6 |
| Akinleye, I. et al., 2011 | No | No | Yes | Yes | No | No | Yes | Yes | Yes | 5 |
| Akyol, M. A. et al., 2020 | Yes | No | Yes | Yes | Yes | Yes | Yes | Yes | Unclear | 7 |
| Al Arifi, M. N., 2020 | Yes | No | Yes | Yes | Yes | Yes | Yes | Yes | Yes | 8 |
| Alacreu, M. et al 2019 | No | No | Yes | Yes | No | Yes | Yes | Yes | No | 5 |
| Algahtani, H. et al 2020 | Yes | Yes | Yes | No | Yes | Yes | Yes | Yes | Unclear | 7 |
| Al-Harbi, A. M. et al., 2022 | Yes | No | Yes | Yes | Yes | Yes | Yes | Yes | Unclear | 7 |
| Alhazzani, A. A. et al., 2020 | Yes | Yes | Yes | Yes | Yes | Yes | Yes | Yes | Unclear | 8 |
| Alhumaidi, F. S. et al., 2020 | Yes | Yes | Yes | Yes | Yes | Yes | Yes | Yes | Yes | 9 |
| Alorfi, N. M. 2022 | Yes | Unclear | Yes | No | No | Yes | Yes | Yes | Unclear | 5 |
| Amado, D. K. and Brucki, S. M. D., 2018 | Yes | No | Yes | Yes | No | Yes | Yes | Yes | Unclear | 6 |
| Anderson, L.N. 2011 | Unclear | No | Yes | Yes | Unclear | Yes | No | Unclear | Yes | 4 |
| Annear, M. J., 2020 | Yes | Yes | Yes | Yes | No | Yes | Yes | Yes | Yes | 8 |
| Arora, D. et al., 2021 | No | Yes | Yes | No | Unclear | Unclear | Yes | Yes | Unclear | 4 |
| Ashworth, R. et al., 2022 | Yes | No | Yes | Yes | Yes | Unclear | Yes | Yes | Unclear | 6 |
| Awuol, M. D. A. et al. 2023 | Yes | No | Yes | Yes | Yes | Yes | Yes | Yes | Yes | 8 |
| Ayalon, L. 2013 | Yes | No | Yes | Yes | Yes | No | No | Yes | Yes | 6 |
| Ayisi-Boateng, N. K. et al., 2022 | Yes | No | No | Yes | Yes | Yes | Yes | Yes | Unclear | 6 |
| Balli, F. N. et al., 2021 | Yes | No | Yes | Yes | Yes | Yes | Yes | Yes | Unclear | 7 |
| Barak, Y. et al., 2018 | Yes | No | No | Yes | Yes | No | Yes | Yes | Yes | 6 |
| Barak, Y. et al., 2020 | Yes | No | Yes | Yes | No | No | Yes | Yes | Yes | 6 |
| Bartlett, L. et al., 2023 | Yes | No | Yes | Yes | Yes | Yes | Yes | Yes | Yes | 8 |
| Breining, A. et al. 2014 | Yes | No | Yes | Yes | Yes | No | Yes | Yes | No | 6 |
| Broche-Perez, Y. et al., 2021 | Yes | No | Yes | Yes | Yes | No | Yes | Yes | Unclear | 6 |
| Cantegreil-Kallen, I. and Pin, S., 2012 | Yes | No | Yes | Yes | Yes | No | Yes | Yes | No | 6 |
| Choi, I. et al.,2022 | Yes | No | No | Yes | Yes | Yes | Yes | Yes | Yes | 7 |
| Crawley, S. et al., 2022 | Yes | No | Yes | Yes | Yes | Yes | Yes | Yes | Unclear | 7 |
| Dai, Y. et al., 2020 | Yes | Yes | Yes | Yes | Yes | Yes | Yes | Yes | Yes | 9 |
| de Krom, F.J.W. et al., 2021 | Yes | No | Yes | Yes | Yes | Yes | Yes | Yes | Unclear | 7 |
| Dukelow, T. et al., 2022 | Yes | No | Yes | Yes | Yes | No | Yes | Yes | Unclear | 6 |
| Elbejjani, M. et al. 2021 | Yes | No | Yes | Yes | Yes | Yes | Yes | Yes | Unclear | 7 |
| El-Gamal, F. et al., 2023 | Yes | No | Yes | No | Yes | Yes | Yes | Yes | Unclear | 6 |
| Eshbaugh, E. M. et al., 2014 | Yes | No | Yes | Yes | Yes | Yes | Yes | Unclear | Unclear | 6 |
| Farrow, M. 2013 | Yes | No | Yes | Yes | Yes | No | Yes | Yes | Unclear | 6 |
| Farrow, M. et al., 2022 | Yes | No | Yes | Yes | No | Unclear | Yes | Yes | Unclear | 5 |
| Fernandez-Fleites, Z. et al., 2021 | Yes | Yes | Yes | Yes | Yes | No | Yes | Yes | Unclear | 7 |
| Friedman, D.B. et al., 2016 | Yes | No | No | Yes | No | No | Yes | Yes | No | 4 |
| Gao, M. X. et al., 2020 | Yes | No | Yes | Yes | Yes | Yes | Yes | Yes | Unclear | 7 |
| Garcia-Ribas, G. et al., 2020 | Yes | No | Yes | Yes | Yes | Yes | Yes | Yes | No | 7 |
| Giebel, C. M. et al., 2019 | Yes | No | Yes | Yes | Yes | No | Yes | Yes | Unclear | 6 |
| Glover, C. M. et al.,2019 | Yes | No | Yes | Yes | Yes | No | Yes | Yes | Unclear | 6 |
| Glynn, R. W. et al., 2017 | Yes | No | Yes | Yes | Yes | Unclear | Yes | Yes | Unclear | 6 |
| Hajek, A. and Konig, H. H., 2020 | Yes | No | Yes | Yes | Yes | No | Yes | Yes | Unclear | 6 |
| Hajek, A. and Konig, H. H., 2020 | Yes | No | Yes | Yes | Yes | No | Yes | Yes | Unclear | 6 |
| Hakami, F. et al., 2023 | Yes | No | Yes | No | Yes | Yes | Yes | Yes | Unclear | 6 |
| Harada, K. et al., 2018 | Yes | No | Yes | Yes | Yes | No | Yes | Yes | No | 6 |
| Heese, K. 2015 | Yes | Yes | Yes | No | Yes | No | No | Yes | Unclear | 5 |
| Heger, I. et al., 2019 | Yes | Yes | Yes | Yes | Yes | Yes | Yes | Yes | No | 8 |
| Heger, I. et al., 2023 | Yes | Yes | Yes | Yes | Yes | Yes | Yes | Yes | No | 8 |
| Hiraki, S. et al., 2009 | Yes | No | Yes | Yes | Yes | No | Yes | Yes | Unclear | 6 |
| Horst, B. R. et al., 2021 | Yes | No | Yes | Yes | No | No | Yes | Yes | Unclear | 5 |
| Howell, J. C. et al., 2016 | Yes | No | Yes | Yes | Yes | Yes | Yes | Yes | Yes | 8 |
| Hudson, J. M. et al. 2016 | Yes | No | Yes | Yes | Yes | Yes | Yes | Yes | No | 7 |
| Imre, N. et al., 2019 | Yes | No | Yes | Yes | Yes | No | Yes | Yes | Unclear | 6 |
| Jang, Y. et al. 2018 | Yes | No | Yes | Yes | Yes | No | Yes | Yes | Unclear | 6 |
| Jernigan, M. et al., 2020 | Yes | No | Yes | Yes | Yes | Yes | Yes | Yes | Unclear | 7 |
| Jonsdottir, M. K. et al., 2022 | Yes | Yes | Yes | Yes | Yes | No | Yes | Yes | No | 7 |
| Joo, S. H. et al., 2021 | Yes | No | Yes | Yes | Yes | Yes | No | Yes | No | 6 |
| Kafadar, A. H. et al., 2021 | Yes | No | Yes | Yes | Yes | Yes | Yes | Yes | Unclear | 7 |
| Keage, H. A. D. et al. 2021 | Yes | No | Yes | Yes | Yes | No | Yes | Yes | Unclear | 6 |
| Kim, J. Y. et al., 2022 | Yes | No | No | Yes | No | Yes | Yes | Yes | Unclear | 5 |
| Kimmel, H. J. et al., 2022 | Yes | No | Yes | Yes | Yes | No | No | Yes | Yes | 6 |
| Kjelvik, G. et al., 2022 | Yes | Yes | Yes | Yes | Yes | Yes | Yes | Yes | Yes | 9 |
| Kwok, T. et al., 2011 | Yes | No | Yes | Yes | Yes | No | Yes | Yes | Yes | 7 |
| LaBond, V. et al., 2014 | Unclear | No | Yes | No | No | No | Yes | Yes | No | 3 |
| Lam, T. P. et al., 2019 | No | No | Yes | Yes | Yes | Yes | Yes | Yes | Yes | 7 |
| Lee, J. and Lim, J. M., 2022 | Yes | Unclear | Yes | Yes | Yes | No | Yes | Yes | Unclear | 6 |
| Lee, S. E. et al., 2010 | Yes | No | Yes | Yes | Yes | Yes | Yes | Yes | Yes | 8 |
| Lee, S. E. et al., 2023 | Yes | No | Yes | Yes | Yes | Yes | No | Yes | Unclear | 6 |
| Lee, S. et al., 2023 | Yes | Yes | Yes | Yes | Yes | Yes | Yes | Yes | Unclear | 8 |
| Lee, Sang E. and Casado, Banghwa Lee, 2019 | Yes | No | Yes | Yes | Yes | Yes | Yes | Yes | Unclear | 7 |
| Li, H. et al., 2022 | Yes | No | Yes | Yes | No | Yes | Yes | Yes | Unclear | 6 |
| Linnenbringer, E. et al., 2010 | Yes | No | Yes | Yes | Yes | No | Yes | Yes | Yes | 7 |
| Liu, D. et al.2019 | Yes | No | Yes | Yes | Yes | Yes | Yes | Yes | Unclear | 7 |
| Liu, D. M. et al.2022 | Yes | No | Yes | Yes | Yes | Yes | Yes | Yes | Yes | 8 |
| Lo, I. L. et al., 2020 | Yes | Yes | Yes | Yes | Yes | Yes | Yes | Yes | Unclear | 8 |
| Low, L. F. et al., 2010 | Yes | Yes | Yes | Yes | Yes | No | Yes | Yes | No | 7 |
| Ma, W. et al.,2022 | Yes | Yes | Yes | Yes | Yes | Yes | Yes | Yes | Unclear | 8 |
| Mansfield, E. et al., 2023 | Unclear | No | Yes | Yes | Yes | No | Yes | Yes | Yes | 6 |
| Marcum, Z. A. et al., 2019 | Yes | No | Yes | Yes | No | No | Yes | Yes | Unclear | 5 |
| Marteau, T. M. et al., 2005 | Yes | No | Yes | Yes | No | No | Yes | Yes | Unclear | 5 |
| Mfene, X. P. and Pillay, B. J., 2023 | Yes | Yes | Yes | Yes | Yes | No | Yes | Yes | Unclear | 7 |
| Milani, S. A. et al., 2020 | Yes | No | Yes | Yes | Yes | Yes | Yes | Yes | Unclear | 7 |
| Mokhtar, S. H. et al., 2021 | No | No | Yes | No | Unclear | No | Yes | Unclear | Unclear | 2 |
| Montepare, J. M.and Pandolfi, G., 2022 | Yes | No | Yes | Yes | Yes | No | Yes | Yes | No | 6 |
| Montiel-Aponte, M. C. and Bertolucci, P. H. F. 2021 | Yes | No | Yes | Yes | Yes | No | Yes | Yes | Unclear | 6 |
| Musoke, P. et al.2021 | Yes | Unclear | Yes | Yes | Yes | Yes | Yes | Yes | Yes | 8 |
| Nagel, A.K. et al., 2021 | No | No | Yes | Yes | Yes | Yes | Yes | Yes | Unclear | 6 |
| Nguyen, T. T., 2023 | Yes | No | Yes | Yes | Yes | Yes | Yes | Yes | Yes | 8 |
| Nielsen, T. R.and Waldemar, G. 2016 | Yes | Yes | Yes | Yes | Yes | No | No | Yes | Yes | 7 |
| Öz, D. et al., 2022 | Yes | Yes | Yes | Yes | Yes | No | Yes | Yes | Unclear | 7 |
| Park, H. Y. et al., 2016 | Yes | Yes | Yes | Yes | Yes | No | Yes | Yes | No | 7 |
| Park, J. S. and Ju, I., 2016 | Yes | No | Yes | Yes | Yes | No | Yes | Yes | No | 6 |
| Parkinson, L. et al.,2022 | No | No | Yes | Yes | Yes | Yes | Yes | Yes | Unclear | 6 |
| Parveen, S. et al., 2022 | Yes | No | Yes | Yes | Yes | Yes | Yes | Yes | Unclear | 7 |
| Paul, P. et al., 2023 | Yes | No | Yes | Yes | Yes | No | Yes | Yes | No | 6 |
| Pipatpiboon, N. et al., 2022 | Yes | Unclear | Yes | Yes | Yes | Yes | Yes | Yes | Unclear | 7 |
| Ramos, H. et al., 2021 | Yes | No | Yes | Yes | Yes | Yes | Yes | Yes | No | 7 |
| Rawlins, J. et al., 2015 | Yes | No | Yes | Yes | Yes | Yes | Yes | Yes | Yes | 8 |
| Riva, M. et al. 2012 | Yes | No | Yes | Yes | Yes | No | Yes | Yes | No | 6 |
| Roberts, J. S. et al., 2014 | Yes | Yes | Yes | Yes | Yes | No | Yes | Yes | Yes | 8 |
| Rosato, M. et al., 2019 | Yes | Yes | Yes | Yes | Yes | No | Yes | Yes | Yes | 8 |
| Seo, H. J. et al., 2015 | Yes | No | Yes | Yes | Yes | Yes | Yes | Yes | Yes | 8 |
| Smith, B.J. et al., 2014 | Yes | Yes | Yes | Yes | Yes | No | Yes | Yes | Yes | 8 |
| Song, D, Yu, D. and Sun, Q. 2022 | Yes | No | Yes | Yes | Unclear | No | Yes | Yes | Unclear | 5 |
| Spittel, S. et al., 2021 | Yes | No | Yes | Yes | Yes | Yes | Yes | Yes | Unclear | 7 |
| Sutin, A. R. et al., 2023 | Yes | Yes | Yes | Yes | Yes | Yes | Yes | Yes | Unclear | 8 |
| Suzuki, R. et al., 2015 | Yes | No | Yes | Yes | Yes | Yes | Yes | Yes | Unclear | 7 |
| Suzuki, Y. et al. 2022 | No | No | No | Yes | Yes | No | Yes | Yes | No | 4 |
| Teichmann, B. et al., 2022 | Yes | No | Yes | Yes | Yes | Yes | Yes | Yes | Unclear | 7 |
| Thanh Nguyen, H. T.and Dinh, D. X. 2023 | Yes | No | Yes | Yes | Yes | No | Yes | Yes | Yes | 7 |
| Van Asbroeck, S. et al., 2021 | Yes | No | Yes | Yes | Yes | No | Yes | Yes | Unclear | 6 |
| Van Patten, R. and Tremont, G. 2020 | Yes | No | Yes | Yes | Yes | No | Yes | Yes | Unclear | 6 |
| Vrijsen, J.; Matulessij, T. F. et al., 2021 | Yes | Yes | Yes | Yes | Yes | No | Yes | Yes | No | 7 |
| Werner, P. et al. 2013 | Yes | Yes | Yes | Yes | Yes | No | Yes | Yes | Yes | 8 |
| Werner, P. et al. 2019 | Yes | Yes | Yes | Yes | Yes | No | No | Yes | Unclear | 6 |
| Werner, P. et al. 2020 | Yes | No | Yes | Yes | Yes | No | Yes | Yes | Unclear | 6 |
| Withers, M. et al. 2019 | Yes | No | Yes | Yes | Yes | Yes | Yes | Yes | No | 7 |
| Wortmann, M. et al., 2010 | Yes | No | Yes | No | Unclear | No | Yes | Unclear | Unclear | 3 |
| Wuttke, A. et al., 2023 | Yes | No | Yes | Yes | Yes | No | Yes | Yes | No | 6 |
| Yamane, N. et al., 2021 | Yes | No | Yes | Yes | Yes | Yes | Yes | Yes | Yes | 8 |
| Yang, H. F. et al., 2015 | Yes | No | No | Yes | Yes | No | Yes | Yes | Unclear | 5 |
| Yun, S. W. et al.,2020 | Unclear | No | Yes | Yes | Yes | Yes | Yes | Yes | Unclear | 6 |
| Zeng, F. et al.,2015 | Yes | Yes | Yes | Yes | Yes | No | Yes | Yes | Unclear | 7 |
| Zeybek, E. and Bektay, M. Y., 2023 | Yes | No | Yes | Yes | Yes | Yes | Yes | Yes | Yes | 8 |
| Zheng, X. et al., 2016 | Yes | No | Yes | Yes | Yes | Yes | Yes | Yes | Yes | 8 |
| Zheng, Y. B. et al., 2020 | No | No | Yes | Yes | Yes | No | Yes | Yes | Unclear | 5 |
| Zheng, Y. B. et al., 2022 | Yes | No | Yes | Yes | Yes | No | Yes | Yes | Unclear | 6 |
| Zulke, A.E. et al., 2022 | No | Yes | Yes | Yes | Unclear | Yes | Yes | Yes | No | 6 |

**Supplemental File S5: JBI Critical Appraisal Checklist for Qualitative Research.^32^**


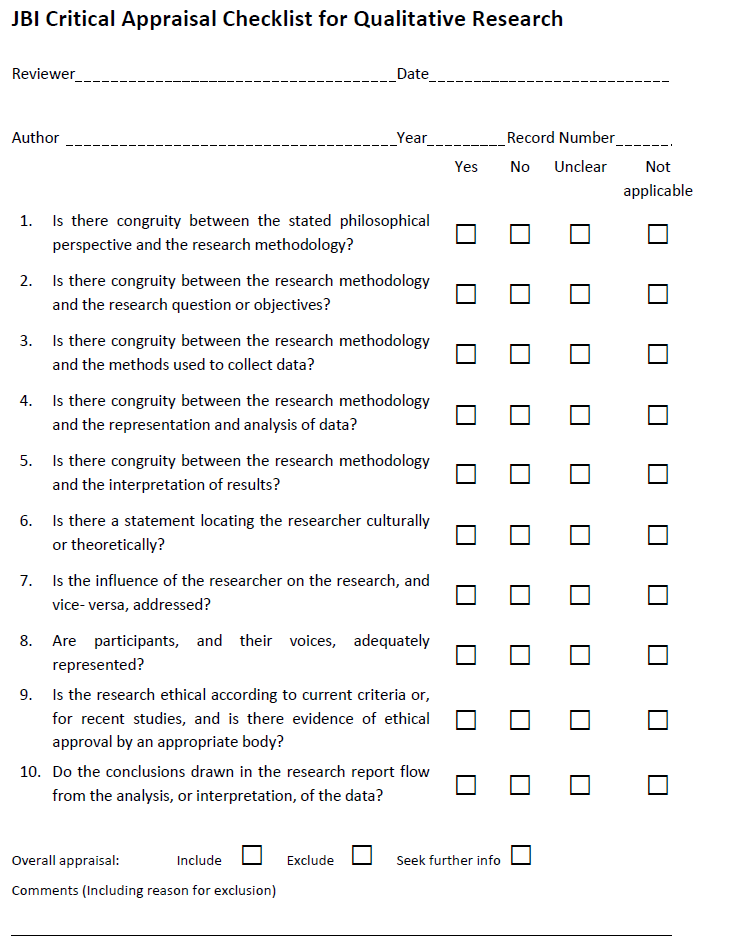


Notes.

Q1: Yes, if philosophical perspective is reported and this perspective matches the research methodology. No, if philosophical perspective is not reported, or of the perspective does not match the research methodology.

Q2: Yes if the methodology used match the question that is being addressed.

Q3: Yes if the methods used match the methodology. For example focus groups conducted because the objective was to explore perceptions of general population adults regarding dementia and its risk factors.

Q4: Yes if the data are analyzed in a way that is in line with the stated methodological position. For example, if an article aims to explore perceptions of dementia and only common perceptions are mentioned while single reported perceptions are not, there is incongruency.

Q5: Yes if the results are interpreted in a way that is appropriate to the methodology. For example if the methodology seeks to understand the meaning of a phenomenon, results cannot be generalized to the total population.

Q6: Yes if a statement is included that clarifies beliefs and values of the researcher, and their potential influence on the study.

Q7: Yes if a statement is included about the relationship between the researcher and the study participants, and the researchers’ own role during data collection.

Q8: Yes if quotes from different participants are included.

Q9: Yes if a statement on the ethical approval process is included.

Q10: Yes if conclusions flow logically from the reported findings.

**Supplemental File S6: Quality assessment of qualitative papers.**

| **Author** | **Q1** | **Q2** | **Q3** | **Q4** | **Q5** | **Q6** | **Q7** | **Q8** | **Q9** | **Q10** | **Overall appraisal** |
| --- | --- | --- | --- | --- | --- | --- | --- | --- | --- | --- | --- |
| Ar & Karanci, 2019 | Yes | Yes | Yes | Yes | Yes | No | No | Yes | Yes | Yes | 8 |
| Balouch, S. et al., 2021 | Unclear | Yes | Yes | Yes | Yes | Yes | Yes | Yes | Yes | Yes | 9 |
| Bridi, L.; Kaki, D. A. et al., 2023 | Yes | Yes | Yes | Yes | Yes | No | No | Yes | Yes | Yes | 8 |
| Cabrera, Laura Y. et al., 2021 | Unclear | Yes | Yes | Yes | Yes | No | No | Yes | Yes | Yes | 7 |
| Eggink, E. 2022 | Unclear | Yes | Yes | Yes | Yes | Yes | Yes | Yes | Yes | Yes | 9 |
| Farina, N. et al., 2020 | Yes | Yes | Yes | Yes | Yes | Yes | Yes | Yes | Yes | Yes | 10 |
| Gubhaju, Lina et al., 2022 | Yes | Yes | Yes | Yes | Yes | Yes | No | Yes | Yes | Yes | 9 |
| Hossain, M. Z. et al., 2019 | Unclear | Yes | Yes | Yes | Yes | No | No | Yes | Yes | Yes | 7 |
| Huisman, M. et al., 2022 | Yes | Yes | Yes | Yes | Yes | No | No | Yes | Yes | Yes | 8 |
| Kim, S. et al. 2015 | Unclear | Yes | Yes | Yes | Yes | Yes | No | Yes | Yes | Yes | 8 |
| Kjelvik, G. et al., 2023 | Yes | Yes | Yes | Yes | Yes | Yes | Yes | Yes | Yes | Yes | 10 |
| McEvoy, C. T. et al., 2023 | Unclear | Yes | Yes | Yes | Yes | Yes | Yes | Yes | Yes | Yes | 9 |
| Olsen, V. et al., 2020 | Unclear | Yes | Yes | Yes | Yes | No | No | Yes | No | Yes | 6 |
| Owokuhaisa, J. et al.,2020 | Unclear | Yes | Yes | Yes | Yes | No | No | Yes | Yes | Yes | 7 |
| Parial, L. L. et al., 2023 | Yes | Yes | Yes | Yes | Yes | Yes | Yes | Yes | Yes | Yes | 10 |
| Price, A. E. et al. 2011 | Unclear | Yes | Yes | Yes | Yes | No | Yes | Yes | Yes | Yes | 8 |
| Pugh, E. et al., 2022 | Unclear | Yes | Yes | Yes | Yes | No | No | Yes | Yes | Yes | 7 |
| Rosenberg, A. et al 2020 | Unclear | Yes | Yes | Yes | Yes | Yes | No | Yes | Yes | Yes | 8 |
| Stansbury, K. L. et al., 2010 | Yes | Yes | Yes | Yes | Yes | Yes | No | Yes | Yes | Yes | 9 |
| Swindells, J. and Gomersall, T., 2020 | Yes | Yes | Yes | Yes | Yes | No | No | Yes | Yes | Yes | 8 |
| Uppal GK et al. 2014 | Unclear | Yes | Yes | Yes | Yes | No | Yes | Yes | Yes | Yes | 8 |
| Vrijsen, J. et al., 2021 | Unclear | Yes | Yes | Yes | Yes | Yes | No | Yes | Yes | Yes | 8 |
| Walsh, S. et al., 2023 | Yes | Yes | Yes | Yes | Yes | Yes | Yes | Yes | Yes | Yes | 10 |
| Yemm, H.; Peel, E. and Brooker, D., 2023 | Unclear | Yes | Yes | Yes | Yes | Yes | Yes | Yes | Yes | Yes | 9 |

**Supplemental File S7: Knowledge of risk and protective factors of dementia.**

| **Risk or protective factor *** | **Number (%) of articles reporting about the risk or protective factor** | **Percentage of participants with knowledge of the risk or protective factor** | | | |
| --- | --- | --- | --- | --- | --- |
|  |  | **Min %** | **Median %** | **IQR %** | **Max %** |
| Demographic factors | | | | | |
| Age | 17 (17.7) | 8.1 | 66.9 | 35.6 – 80.2 | 89.5 |
| Sex | 5 (5.2) | 21.1 | 43.5 | 30.5 – 45.9 | 82.0 |
| Education | 7 (7.3) | 5.0 | 19.5 | 7.8 – 54.9 | 75.4 |
| Vascular and metabolic risk factors | | | | | |
| Obesity | 13 (13.5) | 15.1 | 30.4 | 27.0 – 43.0 | 60.9 |
| Diabetes | 17 (17.7) | 8.5 | 34.5 | 27.6 – 47.0 | 69.2 |
| Dyslipidemia | 32 (33.3) | 6.4 | 39.4 | 28.7 - 56.1 | 86.0 |
| Hypertension | 51 (53.1) | 1.2 | 44.2 | 26.1 – 57.0 | 88.3 |
| Heart disease | 13 (13.5) | 8.0 | 24.8 | 19.0 – 47.0 | 72.9 |
| Stroke | 9 (9.4) | 3.0 | 65.5 | 64.3 – 73.3 | 91.0 |
| Chronic kidney disease | 6 (6.3) | 5.6 | 9.9 | 7.6 – 15.2 | 41.5 |
| Lifestyle factors | | | | | |
| Smoking | 23 (24.0) | 3.1 | 38.5 | 29.3 – 56.9 | 94.0 |
| Excessive alcohol | 26 (27.1) | 5.1 | 45.1 | 31.2 – 64.1 | 89.0 |
| Other substance (ab)use | 1 (1.0) | 22.0 | 22.0 | 22.0 – 22.0 | 22.0 |
| Diet | 27 (28.1) | 4.5 | 48.8 | 34.5 – 65.6 | 91 |
| Food additives | 6 (6.3) | 11.0 | 42.6 | 20.9 – 64.6 | 77.0 |
| Physical activity | 35 (36.5) | 3.8 | 71.5 | 46.9 – 88.3 | 100 |
| Cognitive activity | 48 (50) | 7.1 | 64.5 | 54.6 – 84.1 | 96.7 |
| Social isolation/interaction | 18 (18.8) | 2.3 | 66.6 | 23.7 – 84.0 | 100 |
| Sleep | 8 (8.3) | 2.8 | 48.4 | 27.0 – 75.9 | 90.8 |
| Stress | 17 (17.7) | 1.4 | 63.7 | 45.0 – 76.0 | 84.9 |
| Environmental factors | | | | | |
| Air Pollution | 10 (10.4) | 3.5 | 25.4 | 16.3 – 41.0 | 70.0 |
| Pesticide exposure | 2 (2.1) | 43.0 | 44.4 | 43.7 – 45.0 | 45.7 |
| Genetic predisposition | | | | | |
| Heredity and family history | 33 (34.4) | 10.3 | 54.5 | 32.7 – 73.8 | 95.6 |
| Other factors | | | | | |
| Depression | 16 (16.7) | 1.9 | 36.2 | 21.0 – 69.0 | 79.0 |
| Traumatic Brain Injury | 19 (19.8) | 0.4 | 65.0 | 18.0 – 76.7 | 90.9 |
| Hearing loss | 9 (9.4) | 4.0 | 41.8 | 24.0 – 44.1 | 78.5 |
| Virus/infection | 3 (3.1) | 38.3 | 49.3 | 43.8 – 54.7 | 60.0 |
| Spiritual/ punishment from the gods | 12 (12.5) | 0.4 | 14.3 | 5.6 – 42.0 | 93.0 |

Notes.

The categorization of the risk- and protective factors was inspired by Imre, Balogh.^99^

Min=minimum percentages of participants that indicated the factor to be a risk or protective factor for dementia; max=maximum percentages; IQR = interquartile range, 25^th^ to 75^th^ percentile.

* Factors were selected on the basis of public beliefs rather than established causality per se.

**Supplemental File S8: Comparison of recall and recognition of risk factors in the general population.**

| **Risk factors** | **Smith^*^**  (Australia, 2014) | **Barak**^¥^  (New Zealand, 2020) | **Fernández-Fleites^*^**  (Cuba, 2021) | **Marcum**  (USA, 2022) | **Broche-Pérez^*^**  (Cuba, 2021) | **Giebel**  (UK, 2019) | **Parkinson**  (Australia, 2022) | **Overall recall**  **(median, IQR)** | **Overall recognition**  **(median, IQR)** |
| --- | --- | --- | --- | --- | --- | --- | --- | --- | --- |
| Age | - | - | - | - | - | - | 89.5% | 89.5 (-) | 66.9 (35.6-80.2) |
| Sex | - | - | - | - | - | - | - | - | 43.5 (30.5-45.9) |
| Education | - | - | - | - | - | - | - | - | 19.5 (7.8-54.9) |
| Obesity | - | - | - | - | - | - | - | - | 30.4 (27.0-43.0) |
| Diabetes | - | - | - | - | - | - | - | - | 34.5 (27.6-47.0) |
| Dyslipidaemia | - | - | 14.1% | - | 6.4% | - | - | 10.3 (8.3-12.2) | 39.4 (28.7-56.1) |
| Hypertension | - | - | 17.2% | - | 8.7% | - | 1.4% | 8.7 (5.1-12.9) | 44.2 (26.1-57.0) |
| Heart disease | - | - | - | - | - | - | 30.6% | 30.6 (-) | 24.8 (19.0-47.0) |
| Stroke | - | - | - | - | - | - | 88.2% | 88.2 (-) | 65.5 (64.3-73.3) |
| Kidney disease | - | - | - | - | - | - | - | - | 9.9 (7.6-15.2) |
| Smoking | 3.1% | 6.9% | 29.6% | 65.0% | 24.0% | 16.9% | - | 24.0 (6.9-29.6) | 38.5 (29.3-56.9) |
| Excessive alcohol | 5.1% | 5.6% | 36.7% | 57.9% | 25.8% |  | 7.9% | 16.9 (6.8-31.3) | 45.1 (31.2-64.1) |
| Other substance use | - | - | - | - | - |  | - | 16.9 (6.8-31.3) | 22.0 (-) |
| Diet | 23.3% | 28.2% | 43.2% | 90.7% | 35.3% | 23.1% | 24.7% | 28.2 (24.0-39.3) | 48.8 (34.5-65.6) |
| Vitamins | - | - | 14.8% | - | 11.0% | - | - | 12.9 (11.9-13.9) | 42.6 (20.9-64.6) |
| Physical activity | 31.3% | 47.7% | 46.7% | 94.8% | 31.0%^ǂ^ | 23.7% | 24.7% | 31.3 (27.9-47.2) | 71.5 (46.9-88.3) |
| Cognitive activity | 57.1% | 19.9% | 62.9% | 72.2% | 53.2% | 21.9% | 38.7% | 53.2 (30.3-60.0) | 64.5 (54.6-84.1) |
| Social isolation | 12.1% | 23.6%* | 32.8% | 88.9% | 16.1% |  | 70.6% | 23.6 (19.0.-51.7) | 66.6 (23.7-84.0) |
| Sleep | - | - | 30.7% | 85.7% | 15.9% | 16.3% | 2.8% | 16.3 (15.9-30.7) | 48.4 (27.0-75.9) |
| Stress | - | 6.5% | - | 53.9% | - | - | 74.7 | 53.9 (30.2-64.3) | 63.7 (45.0-76.0) |
| Air pollution | - | - | - | - | - | - | 0.9% | 0.9 (-) | 25.4 (16.3-41.0) |
| Pesticide exposure | - | - | - | - | - | - |  |  | 44.4 (43.7-45.0) |
| Genetics | - | 20.8% | - | - | - | 20.6% | 95.6% | 20.8 (20.7-58.2) | 54.5 (32.7-73.8) |
| Depression | - | 6.5% | - | - | - | - | - | 6.5 (-) | 36.2 (21.0-69.0) |
| Traumatic brain injury | - | 6.5% | - | - | - | 18.1% | 0.9% | 6.5 (3.7-12.3) | 65.0 (18.0-76.7) |
| Hearing loss | - | - | - | - | - | - | - | - | 41.8 (24.0-44.1) |
| Virus or infection | - | - | - | - | - | - | 38.3% | 38.3 (-) | 49.3 (43.8-54.7) |
| Spiritual | - | - | - | - | - | - | - | - | 14.3 (5.6-42.0) |
| Financial difficulties | - | - | - | - | - | 21.9% | - | 21.9 (-) | - |

* applied roughly the same method, with the exception that Smith et al. reported up to five risk factors, whereas the other two studies reported more. Cognitively stimulating leisure activities were scored separately in Cuba from the mental (cognitive) activity; the latter ranked much higher and was used in this table.

¥ enquired about a maximum 3 risk factors and 3 protective factors; social activity/isolation was mentioned among the protective ones by 17.6% and as a risk factor by 23.6%; similarly physical activity/inactivity as protective by 47.7% and as risk factor by 12.0%

ǂ physical activity for the overall group in the article by Broche-Pérez was estimated from the subgroups.

**Supplemental File S9: Meta-regression analysis of all risk and protective factors assessed in at least 20 studies.**

This includes including A) Hypertension, B) Dyslipidaemia, C) Smoking, D) Excessive alcohol, E) Healthy diet, F) Physical Activity, G) Cognitive activity, and H) Heredity.

|  | | **A) Hypertension (N=52)** | | **B) Dyslipidaemia** **(N=33)** | |
| --- | --- | --- | --- | --- | --- |
| **Predictors** | | **Crude model** | **Adjusted model** | **Crude model** | **Adjusted model** |
|  |  | β (95%CI) | β (95%CI) | β (95%CI) | β (95%CI) |
| Age, per 10 years | | 1.56 (-2.71, 5.82) | 3.86 (-0.55, 8.27) | 0.97 (-4.13, 6.07) | 0.32 (-5.74, 6.36) |
| Percentage of women, per 10 units | | 4.39 (-0.59, 9.37) | 2.69 (-1.95, 7.33) | 5.57 (-1.39, 12.53) | 3.67 (-3.19, 10.54) |
| Percentage of high education, per 10 units | | 1.73 (-0.35, 3.82) | 1.68 (-0.94, 4.32) | 1.07 (-1.65, 3.78) | -1.54 (-5.56, 2.47) |
| Quality appraisal score | | 3.07 (-2.29, 8.43) | 2.29 (-2.63, 7.21) | 3.53 (-3.35, 10.42) | 2.44 (-3.94, 8.82) |
| Study Population | Adult | Ref. | Ref. | Ref. | Ref. |
|  | Healthcare | **23.63 (8.17, 39.09)** | 18.28 (-1.19, 37.74) | **19.59 (3.79, 35.40)** | **24.37 (2.60, 46.14)** |
|  | Caregivers & others | **20.44 (7.52, 33.36)** | **18.17 (5.24, 31.11)** | **24.65 (4.85, 44.45)** | **26.03 (5.23, 46.83)** |
| Year of publication | ≤ 2015 | Ref. | Ref. | Ref. | Ref. |
|  | 2016 - 2020 | 16.83 (-14.08, 47.76) | 20.31 (-6.90, 47.52) | 7.43 (-35.69, 50.54) | 34.41 (-11.25, 80.07) |
|  | ≥ 2021 | 20.99 (-9.65, 51.64) | 23.11 (-3.97, 50.19) | 13.48 (-28.97, 55.92) | 40.89 (-5.73, 87.52) |
| Geographical location | Europe | Ref. | Ref. | Ref. | Ref. |
|  | North America | 8.64 (-11.13, 28.41) | 15.84 (-0.79, 32.46) | 13.31 (-8.39, 35.01) | 15.64 (-4.38, 35.65) |
|  | Australia | -8.73 (-34.71, 17.26) | -5.65 (-28.02, 16.70) | **-** | **-** |
|  | South America | -1.51 (-22.67, 19.65) | 2.22 (-16.29, 20.73) | -15.05 (-43.63, 13.51) | -10.21 (-37.12, 16.71) |
|  | Asia | 13.15 (-0.86, 27.16) | **20.76 (8.65, 32.87)** | 12.14 (-2.83, 27.12) | **20.82 (5.96, 35.68)** |
|  | Africa | 16.43 (-9.55, 42.42) | 12.91 (-8.98, 34.81) | **36.35 (7.78, 64.92)** | 22.49 (-5.94, 50.92) |
| Assessment method | Self-administered | Ref. | Ref. | Ref. | Ref. |
|  | Interview | -7.88 (-23.33, 7.55) | -3.83 (-18.99, 11.33) | 1.01 (-24.24, 26.27) | -8.26 (-34.07, 17.56) |
|  | Combined | 12.59 (-17.78, 42.96) | 7.00 (-21.44, 35.44) | 16.11 (-26.25, 58.47) | 20.71 (-17.68, 59.09) |

Estimates represent change in the percentage of participants in a study indicating the risk factor of interest. For age, for percentage of women, and percentage with higher education were rescaled for interpretation by 10% or 10-unit increase. The adjusted model includes the variable of interest along with age, gender, education and study population. The percentage of missing values was 3.03% for percentage of women, 5.05% for age, and 15.15% for percentage of participants with higher education. These missing values were imputed using multivariate imputation by chained equations (MICE) with predictive mean matching.

|  | | **C) Smoking (N=23)** | | **D) Excessive alcohol** **(N=27)** | |
| --- | --- | --- | --- | --- | --- |
| **Predictors** | | **Crude model** | **Adjusted model** | **Crude model** | **Adjusted model** |
|  |  | β (95%CI) | β (95%CI) | β (95%CI) | β (95%CI) |
| Age, per 10 years | | 3.45 (-4.89, 11.80) | 2.55 (-5.91, 11.02) | 6.42 (-1.44, 14.28) | **8.51 (0.08, 16.94)** |
| Percentage of women, per 10 units | | 3.03 (-4.12, 10.18) | 1.44 (-5.85, 8.74) | -6.26 (-12.82, 0.29) | **-8.49 (-15.81, -1.17)** |
| Percentage of high education, per 10 units | | **4.39 (0.50, 8.29)** | 3.96 (-1.32, 9.24) | 1.02 (-2.94, 4.98) | 1.72 (-2.58, 6.01) |
| Quality appraisal score | | -7.70 (-15.93, 0.52) | **-10.18 (-18.55, -1.81)** | -2.10 (-11.21, 6.99) | -2.47 (-11.34, 6.41) |
| Study Population | Adult | Ref. | Ref. | Ref. | Ref. |
|  | Healthcare | 27.53 (-18.58, 73.65) | 2.85 (-53.26, 58.96) | -12.12 (-61.17, 36.93) | 18.40 (-42.11, 78.91) |
|  | Caregivers & others | -5.09 (-33.02, 22.83) | -7.99 (-35.84, 19.86) | -1.29 (-27.37, 24.78) | 2.03 (-22.14, 26.21) |
| Year of publication | ≤ 2015 | Ref. | Ref. | Ref. | Ref. |
|  | 2016 - 2020 | 19.45 (-15.67, 54.58) | 10.16 (-32.99, 3.32) | 7.92 (-29.17, 45.01) | 13.62 (-26.95, 54.19) |
|  | ≥ 2021 | 9.74 (-24.57, 44.05) | -0.08 (-41.03, 40.85) | 2.88 (-33.17, 38.92) | 15.37 (-22.63, 53.36) |
| Geographical location | Europe | Ref. | Ref. | Ref. | Ref. |
|  | North America | 22.08 (-10.05, 54.22) | 16.47 (-25.43, 58.38) | 15.85 (-14.99, 46.69) | 27.89 (-9.88, 65.68) |
|  | Australia | **-23.54 (-46.72, -0.37)** | -24.23 (-59.13, 10.66) | **-30.25 (-53.57, -6.93)** | -24.57 (-54.56, 5.42) |
|  | South America | 1.86 (-23.03, 26.76) | 1.73 (-37.15, 40.61) | -16.48 (-42.55, 9.59) | -12.12 (-45.36, 21.12) |
|  | Asia | 1.19 (-23.71, 26.08) | 0.68 (-28.09, 29.46) | 11.53 (-9.97, 33.03) | 8.67 (-14.20, 31.54) |
|  | Africa | - | - | -17.95 (-59.99, 24.09) | -32.43 (-89.22, 24.36) |
| Assessment method | Self-administered | Ref. | Ref. | Ref. | Ref. |
|  | Interview | -12.57 (-34.96, 9.83) | -0.13 (-32.34, 32.08) | -4.69 (-24.52, 15.12) | -11.78 (-33.07, 9.51) |
|  | Combined | - | - | - | - |

Estimates represent change in the percentage of participants in a study indicating the risk factor of interest. For age, for percentage of women, and percentage with higher education were rescaled for interpretation by 10% or 10-unit increase. The adjusted model includes the variable of interest along with age, gender, education and study population. The percentage of missing values was 3.03% for percentage of women, 5.05% for age, and 15.15% for percentage of participants with higher education. These missing values were imputed using multivariate imputation by chained equations (MICE) with predictive mean matching.

|  | | **E) Healthy diet** **(N=27)** | | **F) Physical activity** **(N=35)** | |
| --- | --- | --- | --- | --- | --- |
| **Predictors** | | **Crude model** | **Adjusted model** | **Crude model** | **Adjusted model** |
|  |  | β (95%CI) | β (95%CI) | β (95%CI) | β (95%CI) |
| Age, per 10 years | | 2.99 (-3.94, 9.92) | 5.90 (-1.98, 13.78) | 1.43 (-6.92, 9.78) | 4.65 (-3.32, 12.62) |
| Percentage of women, per 10 units | | 2.07 (-5.55, 9.68) | 0.35 (-7.60, 8.29) | 0.13 (-7.08, 7.34) | **6.33 (1.84, 10.85)** |
| Percentage of high education, per 10 units | | 3.09 (-0.53, 6.72) | 3.45 (-1.74, 8.64) | **5.16 (1.96, 8.36)** | -1.90 (-8.39, 4.58) |
| Quality appraisal score | | -0.81 (-7.29, 5.67) | -1.94 (-9.55, 5.66) | -1.47 (-8.73, 5.79) | -1.01 (-7.88, 5.87) |
| Study Population | Adult | Ref. | Ref. | Ref. | Ref. |
|  | Healthcare | 17.56 (-17.27, 52.39) | 8.10 (-42.64, 58.85) | 22.97 (-6.39, 52.35) | -6.15 (-45.85, 33.54) |
|  | Caregivers & others | -0.44 (-29.47, 28.59) | -2.59 (-31.88, 26.70) | -22.08 (-55.47, 11.29) | -24.32 (-55.00, 6.37) |
| Year of publication | ≤ 2015 | Ref. | Ref. | Ref. | Ref. |
|  | 2016 - 2020 | -7.56 (-33.84, 18.72) | -21.66 (-50.95, 7.62) | **-3.28 (33.99, 27.43)** | -21.27 (-50.98, 8.43) |
|  | ≥ 2021 | 3.02 (-21.78, 27.81) | -9.57 (-38.48, 19.34) | 4.70 (-22.42, 31.83) | -5.41 (-31.12, 20.31) |
| Geographical location | Europe | Ref. | Ref. | Ref. | Ref. |
|  | North America | **37.48 (16.08, 58.87)** | **36.29 (12.25, 60.34)** | **26.86 (2.16, 51.55)** | **28.54 (3.49, 53.58)** |
|  | Australia | **-25.75 (-43.47, -8.04)** | -25.22 (-51.83, 1.38) | **-28.23 (-47.59, -8.85)** | **-24.90 (-46.81, -3.00)** |
|  | South America | -11.44 (-36.69, 13.81) | -6.67 (-36.79, 23.45) | **-42.70 (-67.39, -18.01)** | -38.92 (-66.57, -11.29) |
|  | Asia | 10.59 (-7.13, 28.30) | 10.74 (-11.39, 32.87) | **20.33 (4.55, 36.11)** | **18.10 (1.66, 34.54)** |
|  | Africa | 16.01 (-18.29, 50.31) | 11.39 (-46.05, 68.83) | - | - |
| Assessment method | Self-administered | Ref. | Ref. | Ref. | Ref. |
|  | Interview | -5.76 (-24.87, 13.36) | 5.91 (-17.51, 29.32) | -15.02 (-38.91, 8.87) | -11.32 (-35.42, 12.79) |
|  | Combined | - | - | - | - |

Estimates represent change in the percentage of participants in a study indicating the risk factor of interest. For age, for percentage of women, and percentage with higher education were rescaled for interpretation by 10% or 10-unit increase. The adjusted model includes the variable of interest along with age, gender, education and study population. The percentage of missing values was 3.03% for percentage of women, 5.05% for age, and 15.15% for percentage of participants with higher education. These missing values were imputed using multivariate imputation by chained equations (MICE) with predictive mean matching.

|  | | **G) Cognitive activity (N=47)** | | **H) Heredity** **(N=35)** | |
| --- | --- | --- | --- | --- | --- |
| **Predictors** | | **Crude model** | **Adjusted model** | **Crude model** | **Adjusted model** |
|  |  | β (95%CI) | β (95%CI) | β (95%CI) | β (95%CI) |
| Age, per 10 years | | 2.01 (-2.95, 6.96) | 4.31 (-1.39, 10.02) | -1.37 (-7.09, 4.35) | -1.33 (-8.06, 5.40) |
| Percentage of women, per 10 units | | -0.33 (-6.29, 5.63) | -2.29 (-8.43, 3.85) | 2.27 (-6.36, 10.89) | 1.89 (-8.11, 11.89) |
| Percentage of high education, per 10 units | | 0.68 (-1.79, 3.15) | 1.42 (-2.19, 5.04) | 0.15 (-2.67, 2.98) | -0.62 (-5.04, 3.81) |
| Quality appraisal score | | 0.49 (-5.05, 6.03) | 0.56 (-5.05, 6.18) | -0.14 (-6.99, 6.71) | -1.06 (-8.46, 6.35) |
| Study Population | Adult | Ref. | Ref. | Ref. | Ref. |
|  | Healthcare | 7.33 (-9.58, 24.24) | 7.25 (-16.36, 30.86) | 7.88 (-11.78, 27.53) | 9.13 (-21.03, 39.29) |
|  | Caregivers & others | -23.83 (-48.63, 0.97) | -25.25 (-50.95, 0.45) | 17.54 (-3.93, 39.02) | 17.17 (-5.79, 40.16) |
| Year of publication | ≤ 2015 | Ref. | Ref. | Ref. | Ref. |
|  | 2016 - 2020 | -1.59 (-23.69, 20.52) | -8.78 (-32.56, 14.99) | -10.48 (-31.19, 10.23) | -6.24 (-29.97, 17.49) |
|  | ≥ 2021 | 13.56 (-6.44, 33.56) | 9.07 (-11.35, 29.48) | 10.75 (-9.31, 30.82) | 14.97 (-8.39, 38.34) |
| Geographical location | Europe | Ref. | Ref. | Ref. | Ref. |
|  | North America | -6.49 (-27.52, 14.54) | -3.49 (-25.27, 18.30) | 10.73 (-15.42, 36.88) | 9.18 (-19.31, 37.68) |
|  | Australia | **-41.06 (-66.92, -15.20)** | **-45.49 (-75.41, -15.57)** | 1.97 (-28.23, 32.16) | -4.59 (-39.42, 30.23) |
|  | South America | -7.62 (-36.68, 21.44) | -8.16 (-38.42, 22.11) | 5.04 (-34.90, 44.98) | -21.27 (-76.26, 33.72) |
|  | Asia | -8.92 (-25.43, 7.59) | -6.51 (-23.45, 10.43) | 6.07 (-16.32, 28.46) | -0.04 (-31.61, 31.51) |
|  | Africa | -3.98 (-38.57, 30.62) | 6.35 (-30.84, 43.53) | 15.39 (-39.04, 69.82) | -2.14 (-77.97, 73.68) |
| Assessment method | Self-administered | Ref. | Ref. | Ref. | Ref. |
|  | Interview | 6.89 (-14.47, 28.26) | 19.31 (-4.61, 43.23) | 3.75 (-18.44, 25.93) | 11.26 (-15.11, 37.63) |
|  | Combined | 21.19 (-28.21, 70.61) | 20.43 (-27.07, 67.93) | -30.88 (-81.06, 19.31) | -27.38 (-80.47, 25.71) |

Estimates represent change in the percentage of participants in a study indicating the risk factor of interest. For age, for percentage of women, and percentage with higher education were rescaled for interpretation by 10% or 10-unit increase. The adjusted model includes the variable of interest along with age, gender, education and study population. The percentage of missing values was 3.03% for percentage of women, 5.05% for age, and 15.15% for percentage of participants with higher education. These missing values were imputed using multivariate imputation by chained equations (MICE) with predictive mean matching.

**Supplemental File S10: Secondary analysis of risk factor knowledge with high-quality articles.**

| **Variable** | **Rosato et al., 2019** | **Smith et al., 2014** | **Dai et al., 2020** | **Alhum-aidi et al., 2020** | **Nielsen & Walde-mar, 2016** | **Werner et al., 2020** | **Kjelvik et al., 2022** | **Roberts et al., 2014** | **Median from high-quality studies** | **Median from all studies** |
| --- | --- | --- | --- | --- | --- | --- | --- | --- | --- | --- |
| Cognitive activity | - | 57.1 | - | 35.3 | - | 62.2 | 84.0 | 92.9 | 62.2 | 64.5 |
| Social isolation | - | 12.1 | - | - | - | - | 80.0 | - | 46.1 | 66.6 |
| Education | - | - | - | - | - | - | - | - | - | 19.5 |
| Diet | 27.7 | 23.3 | - | - | 34.5 | 62.5 | 56.0 | 87.3 | 45.3 | 48.8 |
| Depression | - | - | - | - | - | - | 46.0 | - | 46.0 | 36.2 |
| Excessive alcohol | 32.8 | 5.1 | - | - | 72.0 | 72.5 | 50.0 | - | 50.0 | 45.1 |
| Heredity | 33.8 | - | - | - | 78.3 | - | - | 68.0 | 68.0 | 54.5 |
| TBI | - | - | - | - | - | - | - | - | - | 65 |
| Smoking | 22.7 | 3.1 | - | - | - | 59.4 | 53.0 | - | 37.9 | 38.5 |
| Hearing loss | - | - | - | - | - | - | 18.0 | - | 18.0 | 41.8 |
| Diabetes | - | - | - | - | - | - | 26.0 | - | 26.0 | 34.5 |
| Hypertension | 20.3 | - | 72.9 | 47.0 | - | 47.2 | 31.0 | - | 47.0 | 44.2 |
| Obesity | - | - | - | - | - | 43.6 | 27.0 | - | 35.3 | 30.4 |
| Air pollution | - | - | - | - | - | - | - | - | - | 25.4 |
| Heart disease | - | - | 72.9 | - | - | - | 19.0 | - | 46.0 | 24.8 |
| CKD | - | - | - | - | - | - | 7.0 | - | 7.0 | 9.9 |
| Physical activity | - | 31.3 | - | - | - | 47.1 | 86.0 | 87.8 | 66.6 | 71.7 |
| Stress | - | - | - | - | - | - | - | 55.3 | 55.3 | 63.7 |
| Dyslipidemia | - | - | - | 43.2 | - | 53.0 | 27.0 | - | 43.2 | 39.4 |
| Age | - | - | - | - | 9.0 | - | - | - | 9.0 | 66.9 |
| Stroke | - | - | - | - | 64.3 | - | - | - | 64.3 | 65.5 |
| Sleep | - | - | - | - | - | - | - | - | - | 48.4 |
| Pesticides | - | - | - | - | - | - | - | - | - | 44.4 |
| Virus/ infection | - | - | - | - | 49.3 | - | - | - | 49.3 | 49.3 |
| Spirituality | - | - | - | - | - | - | - | - | - | 14.3 |
| Substance. abuse | - | - | - | - | - | - | - | - | - | 22.0 |
| Sex | - | - | - | - | - | - | - | - | - | 43.5 |
| Food additives | - | - | - | - | - | - | - | 70.7 | 70.7 | 42.6 |

Articles with a ‘yes’ on Q1/Q5, Q Q2, and Q9 of the Critical Appraisal Checklist for studies reporting prevalence data were considered as high-quality articles.

**Supplemental File S11: Funnel plots and Egger’s regression test of all risk and protective factors included in meta-regression.**

**
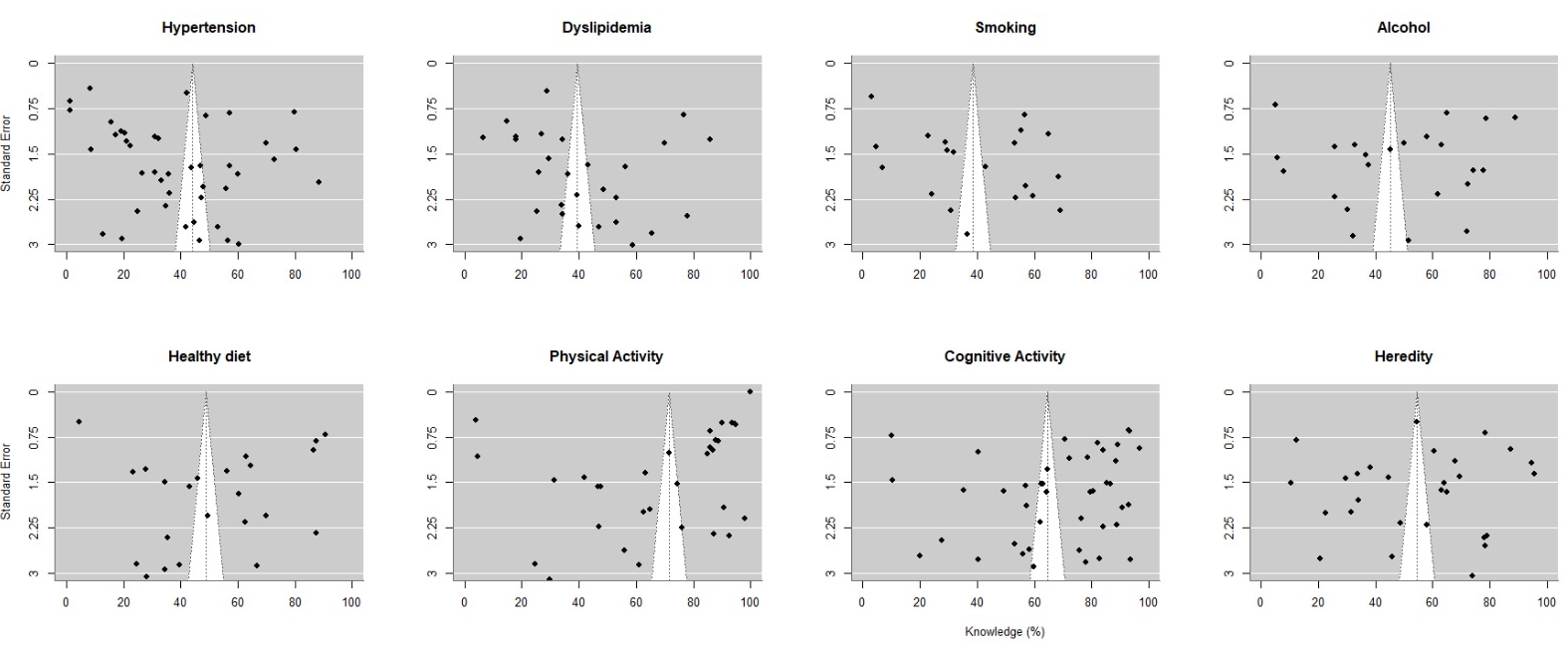
**

| No. | Risk factor | Number of studies | Egger results |
| --- | --- | --- | --- |
| 1 | Hypertension | 52 | t = 2.5555, df = 50, p = **0.0137** |
| 2 | Dyslipidemia | 33 | t = 1.1477, df = 31, p = 0.2599 |
| 3 | Smoking | 23 | t = 2.3455, df = 21, p = **0.0289** |
| 4 | Alcohol | 27 | t = -0.0765, df = 25, p = 0.9397 |
| 5 | Diet | 27 | t = 0.7612, df = 25, p = 0.4536 |
| 6 | Physical activity | 35 | t = -0.7054, df = 32, p = 0.4856 |
| 7 | Cognitive activity | 47 | t = -1.3742, df = 45, p = 0.1762 |
| 8 | Heredity | 35 | t = 0.0499, df = 33, p = 0.9605 |
